# Identification of Tumor Suppressors and Oncogenes from Genomic and Epigenetic Features in Ovarian Cancer

**DOI:** 10.1371/journal.pone.0028503

**Published:** 2011-12-08

**Authors:** Kazimierz O. Wrzeszczynski, Vinay Varadan, James Byrnes, Elena Lum, Sitharthan Kamalakaran, Douglas A. Levine, Nevenka Dimitrova, Michael Q. Zhang, Robert Lucito

**Affiliations:** 1 Bioinformatics and Genomics, Cold Spring Harbor Laboratory, Cold Spring Harbor, New York, United States of America; 2 Philips Research North America, Briarcliff Manor, New York, United States of America; 3 Gynecology Service, Department of Surgery, Memorial Sloan-Kettering Cancer Center, New York, New York, United States of America; 4 Department of Molecular and Cell Biology, The University of Texas at Dallas, Richardson, Texas, United States of America; The University of Hong Kong, China

## Abstract

The identification of genetic and epigenetic alterations from primary tumor cells has become a common method to identify genes critical to the development and progression of cancer. We seek to identify those genetic and epigenetic aberrations that have the most impact on gene function within the tumor. First, we perform a bioinformatic analysis of copy number variation (CNV) and DNA methylation covering the genetic landscape of ovarian cancer tumor cells. We separately examined CNV and DNA methylation for 42 primary serous ovarian cancer samples using MOMA-ROMA assays and 379 tumor samples analyzed by The Cancer Genome Atlas. We have identified 346 genes with significant deletions or amplifications among the tumor samples. Utilizing associated gene expression data we predict 156 genes with altered copy number and correlated changes in expression. Among these genes CCNE1, POP4, UQCRB, PHF20L1 and C19orf2 were identified within both data sets. We were specifically interested in copy number variation as our base genomic property in the prediction of tumor suppressors and oncogenes in the altered ovarian tumor. We therefore identify changes in DNA methylation and expression for all amplified and deleted genes. We statistically define tumor suppressor and oncogenic features for these modalities and perform a correlation analysis with expression. We predicted 611 potential oncogenes and tumor suppressors candidates by integrating these data types. Genes with a strong correlation for methylation dependent expression changes exhibited at varying copy number aberrations include CDCA8, ATAD2, CDKN2A, RAB25, AURKA, BOP1 and EIF2C3. We provide copy number variation and DNA methylation analysis for over 11,500 individual genes covering the genetic landscape of ovarian cancer tumors. We show the extent of genomic and epigenetic alterations for known tumor suppressors and oncogenes and also use these defined features to identify potential ovarian cancer gene candidates.

## Introduction

In the United States, there will be over 22,000 new cases of ovarian cancer in 2011. Of those, approximately 14,000 will succumb to the disease. In order to better treat these women and improve survival, our goal is to determine the molecular changes that have occurred in the patients' tumors, and to be able to interpret the significance these changes have on the growth and development of the tumor. This aberrant growth is a result of chromosomal abnormalities and epigenetic variations [1], [2]. In addition, generally low rates of somatic nucleotide mutation in ovarian cancer as compared to other solid tumors suggest an increased significance of copy number and epigenetic aberrations. This type of regulation has been shown to affect many tumor suppressors and oncogenes pertaining to ovarian cancer [3].

Copy number variations (CNV) are a common occurrence in all forms of cancer [4], [5], [6], [7], [8], [9]. A typical cancer sample exhibits an average of 17% amplifications and 16% deletions within an entire genome. Somatic copy number alterations have been shown to significantly affect pathways involving kinase function, cell cycle regulation, the Myc and NF-κB networks and apoptosis [4]. Detection of these alterations and identification of the specific genes responsible for cancer proliferation can help to molecularly subtype cancers and lead toward more individualized cancer-type specific therapies [7], [10], [11], [12], [13], [14], [15], [16], [17], [18], [19], [20].

Epigenetic properties of the cancer genome correlate with the development and function of the cancer cell [1], [21], [22], [23], [24]. Specifically, DNA methylation at gene promoter regions can regulate the gene expression of various oncogenes and tumor suppressors [25], [26], [27]. It has been proposed that total DNA cytosine 5C-methylation between normal and cancer cells appears to be redistributed to specific CpG loci in the cancer cell [28], [29]. Loss of function or transcriptional silencing via hypermethylation has been identified for tumor suppressor genes, while hypomethylation has been attributed to oncogenesis and the loss of imprinting properties of certain cancer related alleles [1], [30].

Tumor suppressor and oncogene genomic and epigenetic features are highly variable within ovarian cancer [3]. Known tumor suppressors and oncogenes do not equally contribute to the development of the cancer. We hope to identify those genetic and epigenetic aberrations that have the most impact on gene function within the tumor. Many of the current bioinformatics protocols employ only single modal analysis to determine gene function of a particular tumor type. A genome wide approach combining multiple sources of genetic aberration data is necessary for the prediction of possibly consistent and epigenetically integrated pathways that function in tumorigenesis. We performed a broad bioinformatics analysis of copy number variation, expression and epigenetic information to identify potential tumor suppressors and oncogenes associated to serous ovarian cancer. Analyzing 42 independent serous ovarian cancer samples and taking advantage of The Cancer Genome Atlas (TCGA; http://tcga.cancer.gov) [31] data to compare and enhance our protocol, we identify abnormal DNA copy number with correlated changes in methylation and expression for serous ovarian cancer genes. The combination of epigenetic and expression data analysis can possibly provide information specific to the molecular basis of cancer and cancer subtypes and elucidate the genes driving various tumors [28], [32], [33], [34], [35]. Thereby, eventually allowing clinicians to incorporate these types of comprehensive multimodal data analyses into tumor biospecific based diagnostics and pathway directed therapeutics [36].

## Methods

### Patient Samples (MSKCC Data)

Tumor DNA from 42 patients with newly diagnosed, untreated, advanced stage, serous ovarian carcinomas seen at the Memorial Sloan Kettering Cancer Center between the period May 1992–February 2003 were included in this study. The samples were collected under research protocols approved by the Memorial Sloan-Kettering Cancer Center IRB. The study on patient samples and analysis of all sample data complied with the guidelines of the Memorial Sloan-Kettering Cancer Center IRB and was approved by the Memorial Sloan-Kettering Cancer Center IRB. Patients individually provided written informed consent to use their specimens for research purposes. In addition, we used 7 ovarian tissue normal samples obtained from The Cooperative Human Tissue Network, a repository of tissue and tumor material run by the National Institutes of Health. We refer to this patient and normal sample set as the MSKCC data set.

### Copy Number Detection via Representational Oligonucleotide Microarray Analysis (ROMA)

The ROMA protocol as previously outlined [11], [15], [37] was performed on a high-density oligonucleotide array containing ∼85,000 features manufactured by Nimblegen Systems Inc. Briefly, complexity-reduced representations [38] consisting of small (200–1200 bp) fragments, generated by cleavage of DNA samples with the restriction endonuclease BglII, were amplified by adapter-mediated PCR of genomic DNA [11]. DNA samples (2 µg) were labeled either with Cy5-dCTP or Cy3-dCTP using Amersham-Pharmacia MegaPrime labeling kit and competitively hybridized to each other on the same slide [11]. Hybridizations consisted of 35 µL of hybridization solution (37% formamide, 4× SSC, 0.1%SDS, and labeled DNA). Microarry application and hybridization was performed as previously reported [11]. Scanned on an Axon GenePix 4000B scanner using a pixel size of 5 µm and all data was imported into S-Plus 2000 analysis software (Insightful, Seattle, WA). The normalized log ratios from each experiment were averaged per segmentation. We then applied the CBS (Circular Binary Segmentation) algorithm to this data. The CBS segmentation method is the circular binary segmentation algorithm as described in Olshen, AB. et. al. [39]. As in prior analysis, CNV segments are defined as regions of statistically combined probe (marker) intensities calculated by the CBS algorithm [39], [40]. All general analysis and statistics were computed using S-plus, R packages and individual Perl/Python scripts. All ROMA data is MIAME compliant and can be found in the GEO database (http://www.ncbi.nlm.nih.gov/geo/) for the subseries accession number GSE28013.

### Methylation Detection via Representational Oligonucleotide Microarray Analysis (MOMA)

The MOMA protocol was performed as previously described [41], [42]. The MOMA methylation detection array has been performed and validated on cell lines and breast cancer tumor samples. Annotated genomic CpG island locations were obtained from the UCSC genome browser. At the time of the experiment the genome contained 26,219 CpG islands in the range of 200–2000 bp. These CpG island locations were covered by MspI restriction fragmentation. Arrays were manufactured by Nimblegen Systems Inc. using the 390,000 probes format. The CpG island annotation from the human genome build 33 (hg17) was used to design a 50-mer tiling array. The primary restriction endonuclease used is MspI. After the digestion linkers were ligated and the material is cleaned by phenol chloroform, precipitated, centrifuged, and resuspended. The material is divided in two, half being digested by the endonuclease McrBc according to specification by New England Biolabs and the other half being mock digested. Procedures for hybridization and washing were reported previously [41]. The procedure was performed in duplicate with a dye-swap for the second experiment. The labels were swapped between the McrBc treated and mock samples. For each probe, the geometric mean of the ratios (GeoMeanRatio) of McrBc treated and control samples were then calculated per experiment and its associated dye swap. Microarray images were scanned on GenePix 4000B scanner and data extracted using Nimblescan software (Nimblegen Systems Inc). The GeoMeanRatios of all the samples in a data set were then normalized using a quantile normalization method [43]. All general analysis and statistics were computed using S-plus, R packages and individual Perl/Python scripts. All MOMA data is MIAME compliant and be found in the GEO database (http://www.ncbi.nlm.nih.gov/geo/) for the subseries accession number GSE27940.

### Gene Expression Analysis for Human Ovarian Tumor Samples

Gene Expression data was performed using the Affymetrix Human Genome U133A array: GEO platform identifier GPL96. RNA was isolated using the trizol protocol. RNA is converted into cDNA and the double-stranded cDNA is used as the template in an in-vitro transcription reaction containing biotinylated CTP and UTP in addition to the four unmodified ribonucleoside triphosphates. The standard affymetrix protocol is applied. Final signal intensities are processed using the RMA normalization method in the affy package of R Bioconductor 2.5. All array data is MIAME compliant and corresponding CEL files can be found in the GEO database (http://www.ncbi.nlm.nih.gov/geo/) for the subseries accession number GSE27943.

### Cross-modal Analysis of The Cancer Genome Atlas Data (TCGA data)

Copy number variation data for primary ovarian tumors was downloaded from TCGA (http://tcga.cancer.gov/) and CBS [39] data files from the Agilent SurePrint G3 Human 1 M CGH (Comparative Genomic Hybridization) Microarray with the label mskcc.org_OV.CGH-1×1M_G4447A were analyzed. The CBS processed data from the TCGA was then annotated with the UCSC Genome Browser hg18 assembly information to assign copy number variation seg.mean values per gene per sample. For the purpose of studying CNV per gene we limited our data to one complete CBS segment per gene per sample. Therefore, if a gene locus is partially covered by two or more CBS segments per sample we did not include it in our analysis. Only if a complete gene locus was within a sample CBS segment was it included into our analysis. Furthermore, we excluded any CBS segment with an informative (num.info) value of less than 4. Additionally, in order to capture significant CNV we only analyzed samples in 90% of the data excluding 5% of the data closest to a seg.mean of 0 from the positive and negative value distribution. TCGA methylation data was obtained from the jhu-usc.edu_OV.HumanMethylation27.2.lvl-3 data files for each corresponding tumor and normal sample. This is from the Illumina Infinium Human27-methylation assay. A final mean beta value for genes with 2 or more probes was calculated per gene per sample. Finally, the TCGA expression data used for this analysis was from the broad.mit.edu HT_HG-U133A gene expression file for each corresponding tumor and normal sample run on the Affymetrix GeneChip HT Human Genome U133A array. We examined 379 samples from the TCGA that were present at the time of our analysis. Prior to the final submission of our manuscript The Cancer Genome Atlas has made public their preliminary report on ovarian carcinoma [31]. Our use of the TCGA data set is to enhance and compare our tumor suppressor and oncogene discovery protocol that we applied to the MOMA-ROMA (MSKCC) dataset. We acknowledge any similar findings we have made using our protocol on the TCGA dataset with that found in the recent TCGA publication.

### Bioinformatic Analysis of MSKCC Copy Number (ROMA), DNA Methylation (MOMA) and Expression Data

**Figure 1:**
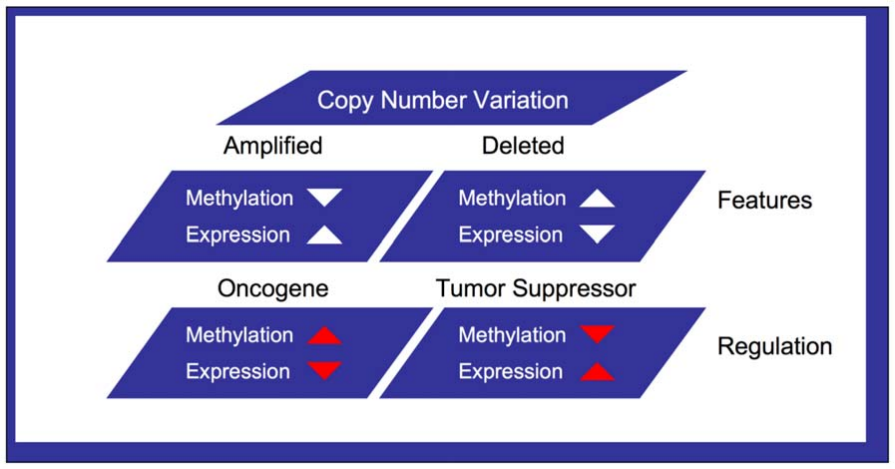
Genomic and epigenetic features of tumor suppressors and oncogenes. Copy number variation is the base genomic feature for our identification of tumor suppressor and oncogenic gene properties in ovarian cancer. An oncogene can be overexpressed under amplified copy number and low methylation, while hypermethylation can be used for regulating expression in a gene amplified state. Similarly, decreased tumor suppressor expression can be the result of partial copy number loss with hypermethylation. Tumor suppressors may also possibly be regulated via hypomethylation in a copy number deleted stated. Our analysis is modeled for such properties and first examines the CNV per gene and then attributes epigenetic alteration for each copy number aberration with gene expression.

All analysis was performed using Perl, Python, Matlab, and R packages. Our strategy was to examine the epigenetic and genomic features for possible tumor suppressors and oncogenes in primary ovarian tumors. With the base feature being copy number variation we examine methylation and expression data for each gene under amplified or deleted copy number conditions. Therefore, an oncogene is classified as an amplified gene having low methylation and elevated expression (Figure 1). This same amplified oncogene may be epigenetically regulated through hypermethylation in ovarian cancer resulting in a decreased expression even if copy number is amplified. Conversely, a tumor suppressor can have lowered copy number variation and be hypermethylated resulting in decreased expression or regulated through hypomethylation allowing for its expression under lowered CNV conditions (Figure 1). ROMA fragments were attributed to genes using the UCSC Genome Browser hg17 assembly. We identified through sample comparison between the TCGA platform and the ROMA platform (for which 7 samples were in common) a ROMA platform-specific threshold of <0.0 seg.mean that captures a maximum percentage of deleted genes while maintaining a minimum false positive percentage of amplified or neutral copy genes. Final gene methylation assignment was performed using the maximum probe value for each MOMA fragment and the maximum MOMA fragment value was attributed to the closest gene. The Wilcoxon signed-rank test was used to calculate enrichment p-values for CNV and expression data and the Benjamini-Hochberg (BH) method was used for the multitest adjustment and False Discovery Rate (FDR) control. Euclidean distances were calculated between normal and tumor samples for methylation and expression data points for all genes in both the MSKCC and TCGA data sets. In the case of the MSKCC data set when sufficient normal sample expression data was not available, a 50× bootstrap sampling was performed using the TCGA normal samples expression data per gene. Single variate and Hotelling multivariate t-tests were performed on these distances to calculate all p-values when performing the methylation and expression analysis at varying copy number values, with statistical multiple test FDR adjustments as above. In order to identify likely functional and pathway changes captured by our feature based gene analysis we tested whether the membership of predicted MSKCC genes in each feature class within a total of 173 KEGG biological pathways was proportional to their size. This translates to identifying pathways whose gene membership in each feature class deviates significantly from the null, as defined by a hypergeometric distribution. The final list of significant pathways was chosen after controlling the false discovery rate by Benjamini-Hochberg multiple testing correction. Data analysis scripts and further analysis information can be found in Analysis S1.

## Results

### Ovarian Tumor Copy Number Aberrations and DNA Methylation

**Figure 2:**
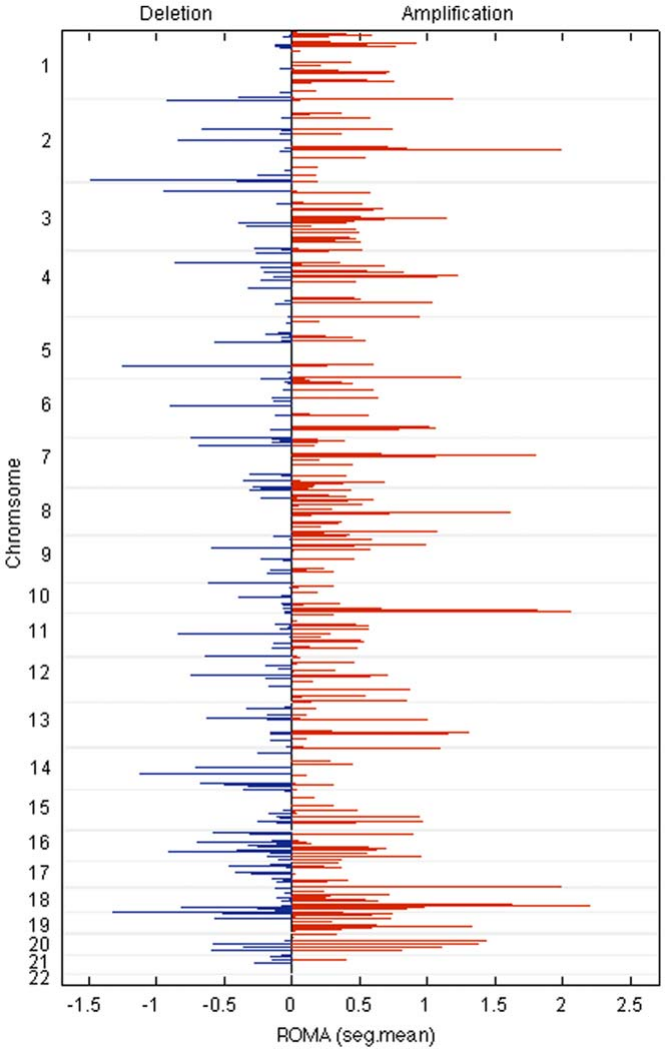
Amplification and deletion breakpoint variability among ROMA segments. Breakpoint positions of copy number variability (deletions depicted in blue, amplifications depicted in red) in 22 chromosomes are shown as determined from ROMA generated segmentation data. The initial altering deletion or amplification genomic position is depicted from all 42 ovarian tumor cancer samples.

**Figure 3:**
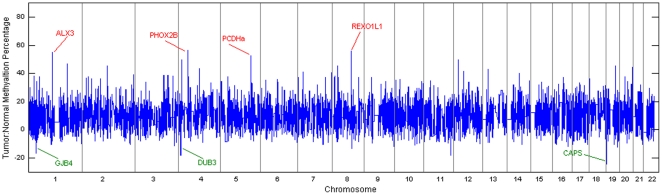
MOMA methylation in ovarian cancer tumors. The tumor:normal ratio percentage for MOMA methylation per gene from 42 ovarian cancer tumor samples and 7 tissue normal samples is outlined per chromosome. For each sample the mean methylation value is calculated from the maximum MOMA value per probe that incorporates the gene promoter region. MOMA methylation data covered 11,978 gene promoter regions. Prominent hypermethylation (red) and hypomethylation (green) genes are labeled and provided in Table S2.

We first individually analyzed both the copy number variation and DNA methylation for each gene by chromosomal position in 42 serous primary ovarian tumors provided by the Gynecology Research Laboratory at Memorial Sloan-Kettering Cancer Center (MSKCC data set) using Representational Oligonucleotide Microarray Analysis (ROMA) [37], [44] and Methylation detection Oligonucleotide Microarray Analysis (MOMA) [41], [42]. The amplified and deleted breakpoint loci cover a total of 561 regions among all samples (Figure 2). ROMA identifies 205 deletion and 356 amplification breakpoints. Breakpoints were defined as regions between each segment (statistically combined probe intensities) calculated using the CBS (circular binary segmentation [39], [40]) method. Among the 42 tumor samples, we find an average of 76 CBS calculated segments per chromosome. Segmentation count per chromosome corresponded with chromosome size except for chromosomes 8, 11, 12, 17, 19, and 20 where segmentation density was greater than normalized for chromosome size and less for chromosomes 6, 9, 10, 14, 15, 16, and 18. The greatest variability of copy number variation (as measured by CBS segmentation mean values) among all samples occurs in chromosomes 19, 2, 10 and 4, respectively (Figure S1). The most frequent deletions (>10% tumor samples) were observed in loci; chr4:q25-q35.2, chr7:p22.3-p15.3, chr8:p23.3-p21.1, chr13q12.11-q34, chr14q32.2-q32.33, chr15q13.3-q21.1, chr16q11.2-q24.3, chr17p13.3-q25.3, chr19:q13.2-q13.43 and chr22:q11.21-q13.33 (Table 1 provides the percentage of all samples deleted within a loci). The most frequently amplified (>10% tumor samples) loci within all chromosomes among all 42 tumor samples are; chr1:p34.4-p34.1, chr1:q21.1-q21.2, chr3:q13.2-q23, chr8:q11.22-q24.3, chr19:q12-q13.12 and chr20:q13.12-q13.2 (Table 1). Three breakpoint symmetry loci (amplifications and deletions at similar genomic positions in multiple samples) were found; chr17:q11.2-q21.32, chr19:q13.12-q13.2 and chr21:q21.3–22.13. Comparing the ROMA results (Table 1) with copy number data of normal individuals found in HapMap [45] shows no overlap with the few amplified regions found in the HapMap normal data set. Overlapping regions of deletion between our CNV results and HapMap are 8p23 and 22q11.23 where both regions show frequent heterozygous loss. We then analyzed the DNA methylation at CpG islands using the same 42 primary ovarian tumors and 7 normal tissue samples (Figure 3). We compiled methylation values for 11,978 gene promoter regions covering 22 chromosomes. When directly compared to normal tissue a total of 68 genes were found to be ranked as hypermethylated and 19 ranked as hypomethylated within 10% of the entire normal to tumor ratio distribution (Table S1). The genes exhibiting methylation values above normal samples include the oncogene PHOX2B, the neuroblastoma associated gene ALX3, the commonly methylated PCDHα gene cluster, POU4F2, REXO1L1, BAPX1, and the potassium-channel KCNJ8. Specifically, REXO1L1, (RNA exonuclease) shows high levels of methylation in both tumor and normal samples however there is a 56% increase of methylation in tumor samples. Genes with the lowest tumor to normal methylation ratios include the chromosome 4 variant of the oncogenic promoting gene ubiquitin hydrolase DUB3 (19% decrease) and CAPS (oncogene implicated in endometrial cancer, 25% decrease). Other hypomethylated genes as compared to normal samples included; RNPC3, USP37, LDHD, GJB4 (gap junction protein), LCN8 (implicated in metastasis) and CGB1 (chorionic gonadotropin, beta polypeptide 1) (Table S1).

**Table 1:** Chromosomal deletions and amplifications in ovarian cancer tumors*.

Location	Frequency
***Deletions***	
**chr4:q25-q35.2**	11%
**chr7:p22.3-p15.3**	12%
**chr8:p23.3-p21.1**	15%
**chr13q12.11-q34**	19%
**chr14q32.2-q32.33**	12%
**chr15q13.3-q21.1**	14%
**chr16q11.2-q24.3**	20%
**chr17p13.3-q25.3**	50%
**chr19:q13.2-q13.43**	20%
**chr22:q11.21-q13.33**	67%
***Amplifications***	
**chr1:p34.4-p34.1**	12%
**chr1:q21.1-q21.2**	19%
**chr3:q13.2-q23**	12%
**chr8:q11.22-q24.3**	35%
**chr19:q12-q13.12**	20%
**chr20:q13.12-q13.2**	15%

*Sample frequency for the most common chromosomal deletions and amplifications found using ROMA in the MSKCC 42 ovarian cancer sample set.

### Correlations of Gene Expression with Copy Number Variation or DNA Methylation

**Figure 4:**
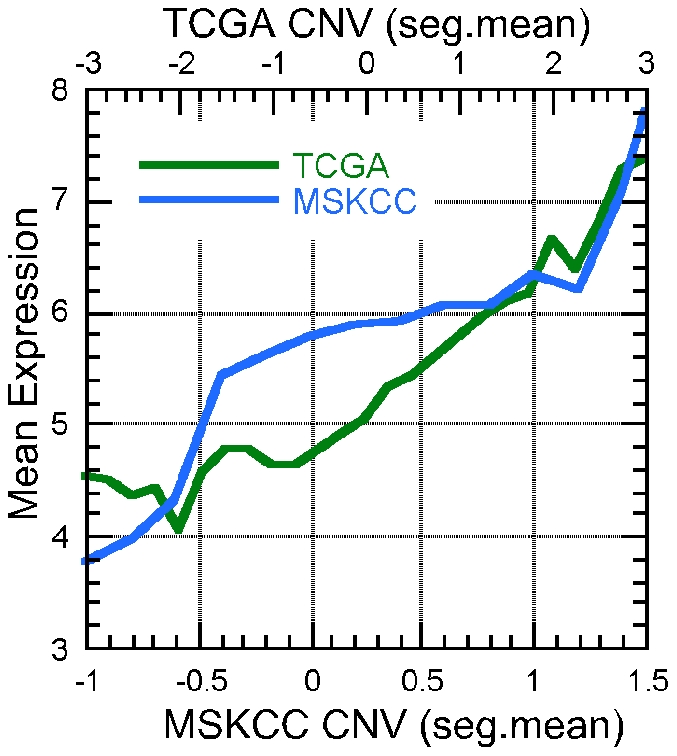
Distribution of gene expression per copy number variation from ovarian cancer tumor cells. As gene copy number variation increases from deletion to amplification the mean gene expression also increases in both the MSKCC (blue line) and TCGA (green line) data sets.

We separately examined the dependency of gene expression (via the Affymetrix Human Genome U133A array, see Methods) on copy number amplification, copy number deletion and promoter methylation in ovarian cancer tumors. We first compared the distribution of gene expression for discrete high and low CNV genes found in our MSKCC data set and TCGA data set. The two data sets showed similar tendencies in expression distribution for genes with high and low copy number variation (Figure 4, Figure S2). As the copy number variation increases from deletion to amplification the average gene expression also increases (Figure 4). We therefore show a correlation between an increase in total gene expression with the amplification of gene copy number in primary ovarian tumors. Additionally, we measured the cumulative distribution of gene expression for deleted and amplified genes. The cumulative distribution is the total percentage of genes found below a dynamic expression threshold. If genes with a low CNV (deleted) are more under expressed than genes with a higher CNV (amplified and over expressed) the cumulative distribution curve results in a steeper rise at lower expression values for deleted genes (indicating a greater percentage of genes found with lower expression values). A maximum cumulative expression difference between 7–17% is observed for genes with low copy number compared to genes with high copy number (Figure S3). Next, we performed expression to CNV correlation per gene for all tumor samples in both the MSKCC data set and TCGA data set. We discovered 124 genes with positive CNV to expression Pearson correlation coefficient limits of ≥0.8 in the TCGA data set (p-values<1.0×10^−10^, Table S2B). The seg.mean amplification and deletion range for the MSKCC data set is not as great as observed in the TCGA data set (Figures S1 and S2) and therefore fewer genes are captured with significant CNV to expression correlations. However, we are able to identify 32 genes with Pearson correlation values≥0.6 (p-values<4.0×10^−5^, Table S2A) with 18 of the 32 genes also identified in the TCGA data set (Table S2A).

Greater gene expression differences between normal and tumor samples are not observed until we rely only on those samples containing genes with extreme amplifications and deletions (Figure 4). Therefore our approach to identify genes with altered copy number variation correlated to expression was to examine the expression values of genes within high and low copy number seg.mean values and compare the expression of those genes to that of normal tissue samples. In an event where there is copy number aberration in normal samples this same type of correlation would be observed. We examined only tumor samples since the magnitude and extent of copy number alterations is more significantly detected through our protocol. Initially we calculated the average expression value for each gene where 20% of the tumor samples showed a CNV value of above 0.50 seg.mean or below −0.50 seg.mean and also filtered out the genes for which the normal expression was not within the standard deviation (the default TCGA CNV thresholds were used which correspond to at least one amplified or deleted copy and with the ability to capture as many altered CNV samples per gene as possible). This strict criteria of 20% of TCGA tumor samples captured 21 genes (Table S3). These 21 genes such as CCNE1 and GSTT1 represent the most altered CNV genes in the tumor samples with differential expression as compared to tissue normal samples in the TCGA data set. However this approach is greatly dependent on the normal gene average expression levels. For TCGA, at the time of our analysis expression information was only available for 8 samples designated as normal. Therefore, a gene such as MYC (most often over expressed in tumor cells) which has a mean sample expression value of 8.93 in the eight TCGA normal samples (Figure S4) and a mean tumor sample expression value of 7.75 (from 339 tumor samples) is not observed by this method. By performing tumor sample specific analysis we may not fully eliminate these variations but hope to limit their magnitude.

So as not to rely on the small normal tissue sampling for expression values, we performed a Wilcoxon rank test only on expression values from a minimum 20% of the tumor samples within very low and high gene copy number seg.mean thresholds. In the TCGA tumor data set this produced a set of 54 genes within a false discovery rate of 5% at seg.mean values of 1.25 and −0.50 for high and low copy number variation, respectively (Table 2, Table S4). The number of genes captured is dependent on the high copy number segmentation mean value used as a filtering threshold (while maintaining a set low copy threshold at −0.50, thereby at minimum capturing a loss of heterozygosity per gene [8]; Figure S5). A total of 1114 genes are captured (FDR<0.05) at a lower CNV threshold of 0.8 seg.mean (Figure S5). With the Wilcoxon rank test we find genes such as MYC, CCNE1, KRAS, NDRG1, MLL4 and MTSS1 for which data set specific normal tissue expression may not be significantly different from all tumor samples but is variable between low and high copy number tumor samples. Conservative threshold limitations of 20% tumor sample inclusion resulted in the identification of genes from extreme CNV loci such as in chromosomes 1, 8, and 19. Of interest, transcription factor CEPBG was found to have good CNV to expression correlation and also expression and methylation correlation in the MSKCC data set. Similarly, performing the Wilcoxon rank test on MSKCC tumor samples at a high copy number threshold ≥0.5 and a low copy number ROMA platform-specific threshold <0.0 (see Methods) we captured 62 genes at a false discovery rate ≤0.05 (Table 2, Table S4). Genes identified in the MSKCC data set were from similar genomic loci as those found in the TCGA data set. Five genes were predicted from both data sets: CCNE1, POP4, UQCRB, PHF20L1 and C19orf2 (Table 2). We have integrated the expression data with CNV to determine the genes that are more likely to be candidates as functioning cancer genes with potential tumor suppressor and oncogenic CNV-expression features. This makes the number of genes in further studies more approachable for functional validation of genes affected by genetic aberrations.

**Table 2:** Selected ovarian cancer genes captured by Wilcoxon rank test based on copy number variation and expression data*.

*Gene Name*	*p-value*	*Chr.*	*Position*	*Gene Function*
***TCGA Data Set***				
***POP4***	*3.53e-16*	*19*	*34789009*	*Component of ribonuclease P.*
***C19orf2***	*1.09e-14*	*19*	*35125264*	*RPB5 binding protein.*
***CCNE1***	*1.56e-14*	*19*	*34995400*	*Cyclin E1, ovarian cancer marker.*
**PAF1**	7.42*e*-10	19	44568109	RNA polymerase II-associated factor.
**KRAS**	3.29*e*-09	12	25249446	GTPase signal transduction.
***UQCRB***	*1.96e-08*	*8*	*97398479*	*Ubiquinol-cytochrome c reductase binding protein.*
**NFKBIB**	7.88*e*-08	19	44082454	NF-κ-B inhibitor.
***PHF20L1***	*1.99e-07*	*8*	*133856785*	*PHD finger protein 20- like 1.*
**CASC1**	1.09*e*-05	12	25152489	Lung adenoma susceptibility 1-like protein.
**NDRG1**	8.11*e*-05	8	134318595	N-myc downstream-regulated gene 1 protein.
**MYC**	1.23*e*-03	8	128817496	Myc proto-oncogene protein, transcription factor.
**MTSS1**	1.91*e*-02	8	125632208	Metastasis suppressor protein 1.
***MSKCC Data Set***				
***POP4***	*4.16e-04*	*19*	*34789009*	*Component of ribonuclease P.*
***CCNE1***	*1.97e-03*	*19*	*34995400*	*Cyclin E1, ovarian cancer marker.*
**FOXJ3**	5.88*e*-03	1	42414796	Forkhead box protein.
**CEBPG**	1.03*e*-02	19	38556448	CCAAT enhancer binding protein.
***C19orf2***	*2.29e-02*	*19*	*35125264*	*RPB5 binding protein.*
***UQCRB***	*2.42e-02*	*8*	*97398479*	*Ubiquinol-cytochrome c reductase binding protein.*
**MYCBP**	2.73*e*-02	1	39101222	c-MYC binding protein.
**MLL4**	2.82*e*-02	19	40900760	Histone-lysine N-methyltransferase.
**STK3**	3.33*e*-02	8	99536036	Serine/threonine-protein kinase 3.
***PHF20L1***	*3.57e-02*	*8*	*133856785*	*PHD finger protein 20- like 1.*
**EBAG9**	4.73*e*-02	8	110621104	Estrogen receptor-binding fragment-cancer associated protein.
**OXR1**	4.76*e*-02	8	107739211	Oxidation resistance protein.

*Results of Wilcoxon Rank test with BH correction of selected genes in ovarian cancer tumor samples. Italics indicate genes captured from both data sets.

We also analyzed the classical dependence of DNA methylation in gene promoter regions with that of gene expression. Methylation data exhibits poorer correlation to expression than copy number variation (Figure S6). We determined Pearson correlations between DNA methylation and gene expression in both the MSKCC and TCGA ovarian primary tumor data sets. Pearson correlation values >0.5 (p-values<2.0×10^−4^, low methylation and high expression to high methylation and low expression) are observed in 86 genes between the two data sets. Prominently, the gene encoding ubiquitin B (UBB) shows a high correlation between methylation and expression in both data sets and RAB25 a known ovarian cancer suspect is also found in the TCGA data set [31], [46] (Tables S2A and S2B).

### Tumor Suppressor and Oncogene Identification Using Methylation and Expression Features Associated with Copy Number Variation

**Figure 5:**
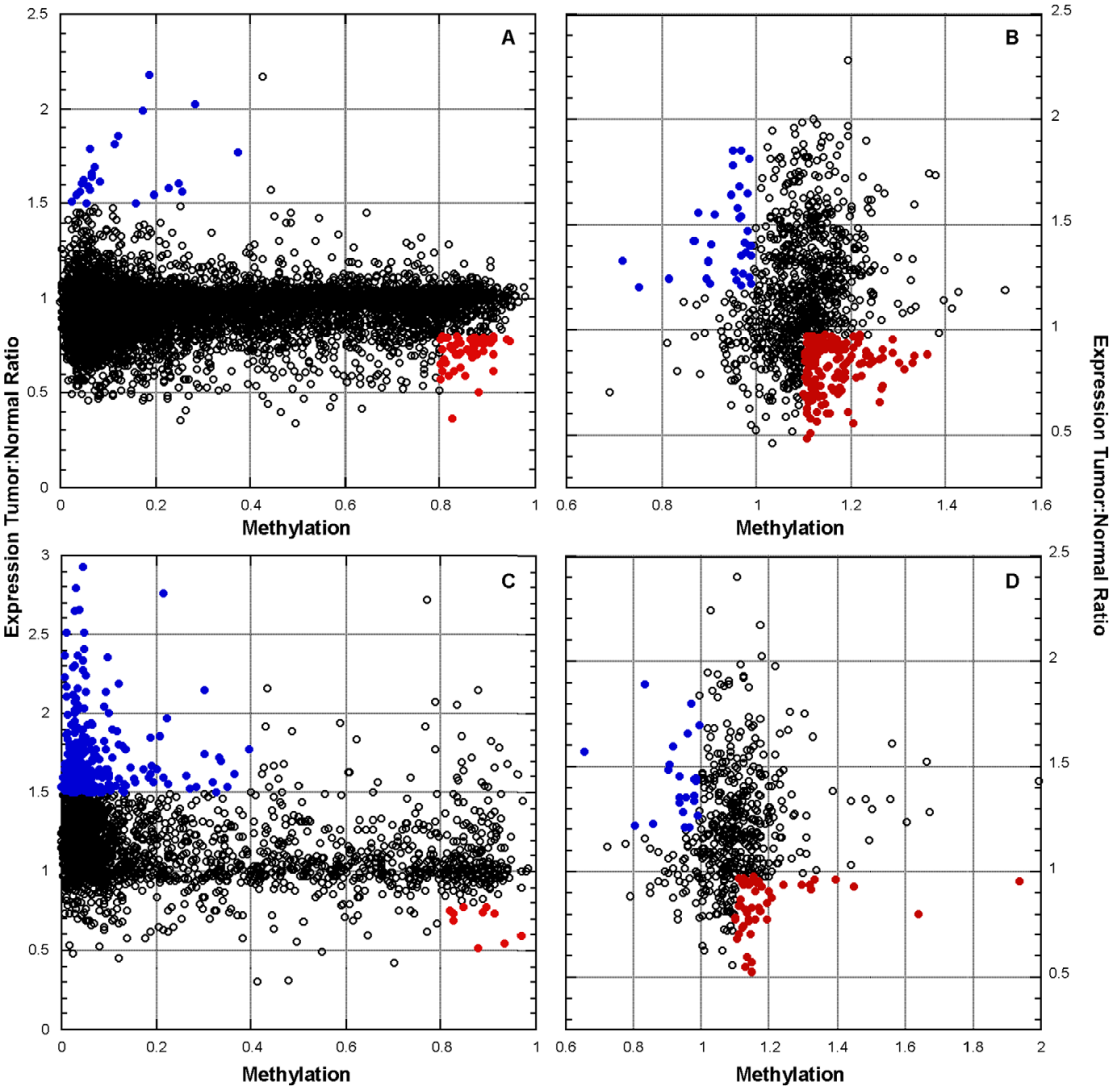
Oncogenic and tumor suppressor features in ovarian cancer. We isolated genes (all points) with extreme copy number variation from the TCGA (**A** and **C**) and MSKCC (**B** and **D**) data sets. Methylation and tumor to normal expression ratio was then compared for genes at low CNV (**A** and **B**) and high CNV (**C** and **D**). Genes with oncogenic features (blue ovals; high expression and low methylation) and tumor suppressor features (red ovals; low expression and high methylation) were identified (Table 3).

We decided to integrate all forms of the data when possible to determine which gene candidates are affected by CNV and methylation and have a concomitant change in gene expression. Correlations with expression will allow us to better determine which gene functions are potentially altered in tumor samples. Methylation and expression gene features can identify potential tumor suppressor and oncogenic behavior in various forms of cancer [3]. Furthermore, this epigenetic significance can be identified when both expression and methylation data types are examined at amplified and deleted CNV changes. Here, we combined methylation and expression data with CNV information from the MSKCC data set and TCGA data set to isolate genes with potential oncogenic and tumor suppressor features (Figure 1). Genes with low CNV or high CNV in the MSKCC and TCGA data sets were filtered and their methylation and expression values identified (Figure 5). In general, a potential tumor suppressor is a gene with suppressed expression and is either functionally altered through mutation, epigenetically silenced or deleted in the cancer cell. Furthermore, tumor suppressors can undergo a dual regulation with one gene copy being deleted and the other regulated via hypermethylation [47]. A potential oncogene can undergo direct or indirect expression control with amplified copy number and/or low methylation features [22], [47], [48]. We looked for genes with such genomic and epigenetic features (Figure 1). We investigated altered expression by tumor sample to normal sample expression ratios (Figure 5, Table S5). Over and under expression thresholds for tumor to normal ratios were determined by the top 25% and bottom 25% of the entire ratio distribution, respectively. Thresholds capturing the extreme 25% distributions within low and high methylation were used for both the MSKCC and TCGA data sets. We isolated 126 genes in the MSKCC data set with tumor suppressor properties of low CNV, low tumor to normal expression ratios and were hypermethylated (Figure 5B). When compared to both the methylation and expression values among the normal data samples, 114 out of these 126 genes had p-values below 5×10^−2^ (results for all genes from this analysis are found in Tables S5, S2A and S2B). The classic tumor suppressor RB1 (retinoblastoma protein, p-value 2×10^−16^) and the tumor suppressor BIK (Bcl-2-interacting killer, apoptosis inducing protein, p-value 1×10^−13^) are among this feature class of gene. A similar analysis with the TCGA data set (Figure 5A) yields 54 genes with potential tumor suppressor behavior among genes with deleted CNV with all but 11 genes having a normal to tumor p-value of<0.05. Examining genes with oncogenic properties such as high tumor to normal expression ratios, high CNV and low methylation we find 33 genes in the MSKCC data set and 285 in the TCGA data set (Figure 5C–D). A total of 611 genes were identified in both data sets using tumor suppressor and oncogenic gene features (Figure 1). Genes previously found as over expressed in ovarian or other cancers were captured as also having over expression and low methylation properties in ovarian cancer. These genes with oncogenic features from either data set were GSK3B, MMP9, ATAD2, MCM2 and UBE2C. Importantly, we discover CDCA8 (member of the chromosomal passenger complex), ATAD2 (AAA family protein implicated in cell proliferation), BOP1 (resides on 8q24 similar to the MYC loci) and EIF2C3 (involved in RNA interference) within both data sets for genes with oncogenic properties of high expression from copy number amplification and low methylation despite low overlapping coverage of genes with all three feature modalities between the MSKCC and TCGA data sets (2703 genes have all three modes of data within both data sets).

**Figure 6:**
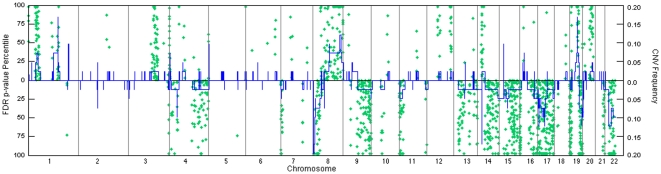
Ranking of significantly expressed and methylated genes with copy number variation. Predicted MSKCC data set genes (green circle) with changes in methylation and expression are overlayed a genome wide stair-plot of ROMA probe sample frequencies per deletion and amplification (blue line). Each predicted gene is percentile ranked according to its FDR p-values (<0.05) between normal and tumor samples.

In addition, we show all MSKCC predicted genes (ranked by percentile by their FDR p-value<0.05) per chromosome with relation to the ROMA probe copy number variation sample frequency (Figure 6). Regions of amplification and deletion are shown along with the number of genes with significant expression and methylation differences from normal. In the MSKCC data set 941 genes were identified with significant changes from normal (based on Euclidean distance measurements as described in Methods) in both DNA methylation and expression in amplified and deleted copy number loci, 25% (238) of which were also discovered using the tumor suppressor and oncogene gene features protocol. Therefore, we illustrate how loci with minor CNV frequency among tumor samples can still contain significantly altered expression and methylation gene features such as seen in chromosomes 2, 6, 10 and 12 (Figure 6). These specific gene identifications within less frequent aberrant loci can potentially lead to a better understanding of direct functional gene contributions in ovarian subtype cancer networks. Finally, in order to identify likely functional and pathway changes captured by our feature based gene analysis we tested the membership of predicted MSKCC genes in each feature class within a total of 173 KEGG biological pathways. Performing a KEGG pathway enrichment analysis on the predicted MSKCC data set genes within each feature class identifies KEGG pathways associated with cancer; endometrial cancer (hsa05213), ErbB signaling pathway (hsa04012), amino acid metabolism (hsa00340), epithelial cell signaling in *h. pylori* infection (hsa0512) and regulation of actin cytoskeleton (hsa4810) (Figure S7).

### Epigenetics and Genomics of Known Tumor Suppressors and Oncogenes

Our Wilcoxon rank analysis revealed correlations for expression and CNV for the oncogenes CCNE1, KRAS, MLL4 and MYC, while our methylation/expression per CNV screen significantly predicts the tumor suppressor RB1. We were therefore curious to understand how all known functional cancer genes were affected by genomic and epigenetic disturbances in ovarian cancer. We analyzed the CNV, methylation and expression data of known tumor suppressors, oncogenes and ovarian cancer biomarkers or cancer related genes. We curated from literature a list of known genes implicated in ovarian cancer or shown to be significant in cancer pathogenesis [3], [4], [7], [16], [23], [31], [49]. We then compiled the methylation and expression properties per copy number variation for each of these genes (Table 3 presents MSKCC data, Tables S2A, S2B and S6 show all data). As suspected, many genes exhibit varying expression values among the three states of copy number variation (amplified, deleted or no change which we term neutral – Table 3, Table S6). Correlating changes of CNV and expression can be seen in genes of the ovarian cancer 19q amplicon [50] CCNE1 (r = 0.73) and AKT2 (r = 0.66), suggesting functional variation within the ovarian cancer sample population. Again, significant deletion frequency of RB1 (67%–83%) is observed in both the TCGA and MSKCC data set, respectively. Furthermore, the tumor suppressors PTEN, TP53, DAB2, CDKN2A, PLAGL1, PEG3, RPS6KA2, NF1, BRCA1, BRCA2 and WWOX, all show ≥50% deletion among primary ovarian tumor samples. Amplification of known oncogenes was less profound however MYC, PIK3CA, ETV6, AURKA, EIF5A2, NOTCH3, and KRAS exhibit ≥20% sample frequency. BRCA1 and BRCA2 show unvarying methylation and expression results at all copy number variation thresholds, plus methylation and expression levels are consistent with normal tissue samples. CDC25A shows approximately 30% deletion among ovarian tumor samples with unvarying methylation levels and expression correlation with deletion. Methylation and expression for the tumor suppressor CDKN2A is highly correlated (r = 0.79) and exhibits significant p-values (<1.0×10^−15^) over the range of copy number variation. A concomitant loss of expression with both methylation and copy number variation is also observed for CDKN2A. When examining oncogenes we see the methylation/expression correlation with CNV for NOTCH3 (p-value<7.5×10^−6^; the p-values are determined from Euclidean distance values of methylation and expression between normal and tumor as described in methods) RAB25 (p-value <5.4×10^−3^), and AURKA (p-value<1.2×10^−10^). For all three genes methylation decreases and expression increases with increased copy number. Finally, looking at cancer related genes, we find a distinct correlation between copy number variation and expression for MLH1 (r = 0.563) and separately the isocitrate dehydrogenase genes. MLH1, a component of the DNA mismatch repair complex, is hypomethylated in samples with deleted CNV suggesting an epigenetic regulation mechanism to increase expression within a loss in gene copy. IDH1 and IDH2, as well as the IDH3 isoforms show positive correlations between expression and copy number variation, ranging from r = 0.732 for IDH2 to r = 0.504 for IDH1. We also see differences in methylation (p-values<6.31×10^−7^) between tumor and normal samples for all IDH genes, as previously reported in glioblastoma [33]. However only IDH3B shows significant differences (p-values<7.2×10^−10^) for methylation and expression at varying copy number. We suggest that expression changes in the IDH genes in ovarian cancer can result from a contribution by copy number variation rather than strictly promoter methylation changes.

**Table 3:** Copy number variation derived methylation and expression of tumor suppressors and oncogenes in ovarian cancer*.

*MSKCC Data Set*					
*Gene*	*Copy Number Variation*	*CNV Frequency*	*Seg.mean*	*Methylation*	*Expression*
***Tumor Suppressors***					
**RB1**	Amplified	0.00	0.00	0.00	0.00
**RB1**	Neutral	0.17	0.03	0.98	5.04
**RB1**	Deleted	0.83	−0.12	0.99	4.66
**PTEN**	Amplified	0.00	0.00	0.00	0.00
**PTEN**	Neutral	0.42	0.03	1.03	5.43
**PTEN**	Deleted	0.58	−0.04	1.04	5.32
**TP53**	Amplified	0.00	0.00	0.00	0.00
**TP53**	Neutral	0.07	0.06	0.97	5.33
**TP53**	Deleted	0.93	−0.10	0.90	4.89
**PLAGL1**	Amplified	0.03	1.06	1.13	10.36
**PLAGL1**	Neutral	0.20	0.04	1.14	5.73
**PLAGL1**	Deleted	0.77	−0.05	1.14	4.94
***Oncogenes***					
**MYC**	Amplified	0.13	0.69	0.96	8.28
**MYC**	Neutral	0.80	0.16	0.98	7.61
**MYC**	Deleted	0.07	−0.04	0.98	8.26
**AKT2**	Amplified	0.03	0.56	1.09	4.93
**AKT2**	Neutral	0.45	0.06	1.09	4.51
**AKT2**	Deleted	0.52	−0.09	1.07	4.50
**FGFR1**	Amplified	0.02	0.60	1.25	6.83
**FGFR1**	Neutral	0.60	0.09	1.12	6.44
**FGFR1**	Deleted	0.38	−0.12	1.13	6.09
**CCNE1**	Amplified	0.12	0.68	1.17	8.44
**CCNE1**	Neutral	0.42	0.09	1.14	7.59
**CCNE1**	Deleted	0.45	−0.05	1.11	6.72
***Biomarkers/Cancer-Related***					
**WFDC2**	Amplified	0.05	0.56	0.94	10.30
**WFDC2**	Neutral	0.70	0.11	0.97	11.21
**WFDC2**	Deleted	0.25	−0.03	0.98	9.55
**PIAS3**	Amplified	0.03	0.86	1.08	6.75
**PIAS3**	Neutral	0.76	0.07	1.11	5.95
**PIAS3**	Deleted	0.21	−0.02	1.17	5.72
**IDH3B**	Amplified	0.00	0.00	0.00	0.00
**IDH3B**	Neutral	0.72	0.08	0.99	7.10
**IDH3B**	Deleted	0.28	−0.04	1.01	6.91
**IDH3G**	Amplified	0.03	0.50	0.97	6.83
**IDH3G**	Neutral	0.82	0.16	0.97	6.98
**IDH3G**	Deleted	0.15	−0.03	1.00	6.51

*Selected ovarian cancer related tumor suppressor and oncogene epigenetic data is presented from the MSKCC data set. The frequency of each gene found in an amplified, neutral or deleted state based on CNV thresholds is provided. The seg.mean threshold for amplified was set at ≥0.50 and for deleted at <0.00. Neutral was defined as not within the amplified or deleted thresholds. And the average seg.mean, methylation and expression values for those CNV states is shown. A complete table with the full summary from both data sets is present in Table S6.

## Discussion

DNA copy number alterations are a common occurrence in all cancers. Specific chromosomal regions and focal points favor either gains or losses in DNA among cancer types [4], [7]. These amplifications and deletions are shown to include tumor suppressors and oncogenes. In addition, DNA methylation exhibits redistribution within a cancer genome [20], [22], [29]. Often times the copy number and DNA methylation profiles are generated as a static representation of a particular cancer's whole genome aberrations. However, the amplitude of specific gene function within ovarian cancer is often highly variable between tumor samples [3]. It is therefore essential to accurately determine each gene's individual functional state within its cancer environment. Here, we not only looked at whole genome patterns of copy number aberrations and methylation but also focus on sample specific CNV and methylation properties for altered genes to provide a better understanding of ovarian cancer gene functionality. We first separately present data of DNA structural variation and DNA methylation changes in ovarian tumors and then combine the two modalities with expression data to identify how these aberrations may affect individual gene function within the tumor population.

We first analyzed the DNA copy number variation of primary ovarian tumors from 42 individuals and compared our findings to The Cancer Genome Atlas data set for ovarian cancer [31]. In analysis of CNV segmentation changes in our 42 tumor samples, DNA variability is shown to be most prevalent in chromosomes 1, 2, 4, 8, 9 and 19. We have shown a large variability in amplification and deletion breakpoint loci in ovarian tumors and identified chromosomal areas of frequent copy number variations. We see a high level of amplification frequency in known oncogenic regions containing MYC (chromosome 8), CCNE1 (chromosome 19) and frequent deletions are found in chromosome arms 4q, 16q, and 17p [16], [51]. Similarly to the TCGA data analysis ROMA detected high frequency copy losses in PTEN, RB1, and NF1. We also show the known but previously unreported in primary ovarian tumors amplification of the MCL region of chromosome 1q21.1-q21.2 [4], [31] and previously unreported deletions in the IDH2 and IDH3 region of chromosome 15. A total of 983 genes are included in amplified and deleted regions. A strong correlation of expression with copy number variation has been reported in ovarian cancer [52]. Here, we primarily focused on quantifying this correlation at varying CNV levels for the purposes of functional annotation. Most significant changes of expression occur at extreme CNV. We show that copy number variation has a strong effect on expression in primary ovarian tumors for 156 genes. Notable genes with correlated CNV and expression include MYC, CCNE1, KRAS, NDRG1, MLL4, MTSS1, C11orf30, MLH1 and CEPBG. Genes identified in the MSKCC data set were from similar genomic loci as those found in the TCGA data set. Five genes were predicted from both data sets: CCNE1, POP4, UQCRB, PHF20L1 and C19orf2. In addition isocitrate dehydrogenase isoforms IDH2 (chr15), IDH3A (chr15), and IDH3B (chr20) show correlated expression to CNV. The IDH1 and IDH2 genes are mutated in glioblastoma and AML cancer patients. IDH1 has been implicated as a prognosis positive biomarker in glioblastoma and AML IDH1/2 mutants show hypermethylation in comparison to other AML subtypes [53], [54]. All the isocitrate dehydrogenase genes exhibit deleted CNV in ovarian cancer samples (ranging from 14%–52%, Table 3). Here, we show for both the IDH2 and IDH3A/IDH3B genes expression is mainly correlated in tumor samples exhibiting deleted copy number to normal copy number.

Next, we examined genome wide DNA methylation in ovarian tumors. DNA methylation alterations are a significant feature of the cancer genome [1], [23]. The protocadherin gene cluster of chromosome 5 has been shown to be frequently hypermethylated and silenced in various forms of cancer [55], [56]. We see broad hypermethylation for both the PCDHα and PCDHβ loci in ovarian cancer as well. We also observe increased levels of methylation in other cancer related genes e.g. ALX3 and PHOX2B both implicated in neuroblastoma [57], [58]. Decreased levels of methylation are seen in genes such as calcyphosin which exhibits oncogenic properties in endometrial cancer [59], lactate dehydrogenase which when inhibited impairs cell proliferation via the Warburg effect of aerobic glycolysis in cancer cells [60] and DUB3, the CDC25A stabilizing protein ubiquitin hyrdolase [61] which has been shown to rescue CDC25A from proteasomal degradation and promote an oncogenic induction response [62]. Hypomethylation with subsequent cellular rise of DUB3 can therefore be a candidate for the cellular regulation of CDC25A protein levels and CDC25A linked oncogenesis.

Finally, to identify genes with a more direct genomic and epigenetic effect on the function of the cancer cell, we directed our focus on the combined gene features of copy number, methylation and expression. Tumor suppressors and oncogenes are often implicated by their transcriptional abnormalities in the cancer cell. It is of interest to understand which tumor suppressors and oncogenes play a direct role in a particular cancer among all genes affected by genomic aberrations. A certain tumor suppressor or oncogene function may be gained or silenced at varying frequencies by different epigenetic and genomic conditions within the tumor sample population. Examining these properties and their affects on gene expression can provide better insight into identifying which genes are most responsible to the pathology of the tumor. We therefore formulate a set of predictive features based on genomic and epigenetic properties of the tumor that can be indicative of altered function for tumor suppressors and oncogenes in the cancer genome (Figure 1). Low expression of various tumor suppressors in cancer cells can be a result of deleted copy number or silencing by promoter hypermethylation. While amplification and promoter hypomethylation can play a role in the over expression of oncogenes. Conversely, a particular known oncogene may be deleted in a particular cancer lessening its pathogenic role within that cancer or an individual sample. Interestingly, for highly amplified genes, a high level of methylation accompanied by low expression could indicate altered tumor suppressor function in the cancer cell. In highly amplified genes, low level methylation and high expression would indicate oncogenic features in a cancer cell. We therefore utilized samples with extreme copy number variations and examined the methylation and expression changes of genes within these aberrant loci to identify potential tumor suppressors and oncogenes. Examining both our primary MSKCC tumor sample data set and the TCGA data set, we discovered 180 genes with tumor suppressor features of low expression with copy number deletion and high methylation. These features are characteristic of known classic tumor suppressors among which the established tumor suppressor RB1 (retinoblastoma protein) was captured. Additionally we find another 48 genes with elevated copy number but low expression and high methylation. For oncogenic epigenetic gene features we discover 318 genes within amplified loci and 65 within deleted copy number loci between the two data sets. Several genes discovered in ovarian cancer tumors with these specific tumor suppressor and oncogenic features have been previously implicated in other cancers and are now shown to have additional methylation and copy number variation properties. Furthermore, 25% of the genes captured with tumor suppressor and oncogenic gene features were represented in 941 MSKCC data set genes (Figure 6) with significant changes in methylation and expression per CNV. Seven genes were identified from both the MSKCC and TCGA data sets that contained strong correlations for methylation dependent expression exhibited at varying copy number aberrations; CDCA8, ATAD2, CDKN2A, RAB25, AURKA, BOP1 and EIF2C3. Four of these seven genes (CDCA8, ATAD2, CDKN2A, AURKA) have direct functional relationships of binding and regulation with other experimentally established oncogenes and tumor suppressors such as TP53, RB1, MYC and E2F1 [63], [64], [65], [66]. Thereby indicating a potential functional cancer module (Figure S8) that can be further computationally and experimentally targeted. Using genomic features specific to aberrations found in tumor sample data captures previously identified tumor suppressors and oncogenes in addition to genes associated with these biomarkers. This genomic and epigenetic function-based feature approach identified genes in cancer pathways such as endometrial cancer, ErbB signaling pathways, epithelial cell signaling and actin cytoskeleton regulation. This type of primary gene function identification approach can provide a base feature set for further machine-learning cancer network prediction protocols.

In addition, cancer genes exhibiting contradictory tumor suppressor or oncogenic epigenetic features in ovarian cancer may provide clues into the regulatory pathways within ovarian cancer. Of note, predicted within the MSKCC data set tumor suppressor features is the established oncogenic transcription factor STAT3 [67], [68], [69]. Here we see significant STAT3 deletion (≥73% sample frequency) contributing to a potential heterozygous gene copy loss in both the TCGA and MSKCC data sets. Furthermore, within samples containing a low copy number of STAT3 gene, slightly higher methylation and lower expression values are observed. This may suggest a decreased role for STAT3 in the oncogenic function within ovarian tumors. Therefore, epigenetic and genomic specific gene features are at the strength of our predictions and can be used to *i*) predict novel gene functions in ovarian cancer and *ii*) elucidate or verify the direct cancer functioning role for previously implicated tumor suppressors or oncogenes. We therefore decided to examine many known cancer oncogenes and tumor suppressors for varying levels of regulation among tumor samples. For instance, the ovarian cancer oncogenes CCNE1 and RAB25 [3], [70] show significant methylation and expression correlation for both amplified and deleted copy number aberrations. The expression levels of these cancer functioning genes differs between samples and the modes of epigenetic regulation exhibit different levels of frequency [3]. Each gene affecting the growth of the tumor is not evenly implicated in all samples. We therefore attempted to illustrate these genomic and epigenetic sample irregularities (such as observed in PLAGL1, CCNE1 and PIAS3) for many of the known ovarian cancer genes (Table 3, Tables S2 and S6). Sample specific feature analysis of identical gene combinations and modules at amplified, neutral or deleted copy number with corresponding epigenetic regulatory features can be used to identify ovarian cancer heterogeneity and the driving genes contributing it. Application of this gene function diversity can be further studied using clinical information for each sample, thereby combining cancer gene modules with each samples' clinical features. The development of this type of knowledge base of gene features in a cancer population will better help identify subtype specific tumor function.

The continuing increase of experimental epigenetic data from various tumor samples offers the ability to computationally search for putative genes with properties in the proliferation of cancer cells. Here we performed a coarse-grained bioinformatics whole genome evaluation of epigenetic features in ovarian cancer tumor cell from two separate platforms covering over 11,500 genes. We demonstrate ovarian cancer specific epigenetic regulation of previously identified cancer genes and cancer biomarkers. Furthermore, we were also able to implicate genes with tumor suppressor and oncogenic epigenetic properties specific to ovarian cancer tumors that have not been previously reported. Examination of multiple cancer epigenetic modalities will help segregate cancer specific genes from randomly altered cancer genes and can possibly elucidate the genetic mediators of ovarian tumorigenesis. The focus on gene combinations with specific copy number aberrations per individual tumor sample plus their methylation and expression properties within those samples allows for the better understanding and eventual identification of tumor type specific cancer pathways.

## Supporting Information

Figure S1 **Copy number variability in ovarian cancer tumor samples.** **A**) Variability per chromosome of all ROMA derived CBS segmentation values for 42 tumor samples in the MSKCC data set is shown. **B**) The mean value (horizontal straight bar) of CNV segmentation values per chromosome and standard deviation (error bars) from 42 tumor samples.
(TIF)

Figure S2 **Copy number variation in TCGA and ROMA samples for ovarian cancer specific genes.** Presented are four examples of copy number variation analyzed per gene (see methods) from TCGA tumor CNV data (open grey circles) and from MSKCC data set ROMA array tumor samples (filled black boxes). CBS segmentation mean (Seg.Mean) values per sample are plotted for four known ovarian cancer significant genes. Amplification and deletion sample comparisons between TCGA and ROMA segmentations are shown for **A**) MYC, **B**) TP53, **C**) CCNE1 and **D**) NCOA3.
(TIF)

Figure S3 **Cumulative distribution of gene expression per copy number variation.** The cumulative distribution function (Fn(*x*) = P(X≤*x*)) for expression is plotted for genes with high (red line) and low (green line) copy number variation discovered from ROMA analysis (A) and found in the TCGA data set (B). Maximum difference in expression distribution between low and high copy is 7% in the TCGA data set and 17% in the MSKCC data set.
(TIF)

Figure S4 **Expression of the MYC gene in ovarian tumor and normal samples in the TCGA data set.** The expression of the gene MYC in ovarian tumor samples and normal samples as identified in the TCGA data set. Expression values are shown for samples with amplified CNV for MYC (left panel), deleted CNV for MYC (center panel) and for normal tissue (right panel). Colored bar shows the expression mean for each condition.
(TIF)

Figure S5 **Genes captured by Wilcoxon rank test in ovarian cancer tumor samples.** The Wilcoxon Rank test was performed on the ovarian cancer tumor TCGA data set. The test ranked expression levels of genes among samples with high and low copy number gene values. With a low CNV seg.mean threshold set at −0.50, the total genes captured was dependent on the high CNV threshold. Shown are the total genes captured (filled in square, dotted line) and number of genes with a FDR<0.50 (filled in circle, solid line) by the Wilcoxon rank test at CNV values of 0.80, 1.0 and 1.25. A total of 54 to 1114 genes with FDR<0.50 is identified using CNV threshold values of 0.80 to 1.25.
(TIF)

Figure S6 **Expression correlation with copy number variation or methylation.** Correlation value distribution per gene of expression to copy number variation (CNV-Expression, green line) and methylation (Methylation-Expression, red line) are shown as a proportion of total genes analyzed for TCGA (**A**) and ROMA-MOMA MSKCC data (**B**).
(TIF)

Figure S7 **KEGG pathway enrichment analysis.** A KEGG pathway enrichment analysis defined by hypergeometric distribution was performed on the genes predicted in the MSKCC data set for each genomic and epigenetic feature class for oncogenes and tumor suppressors. Amp. abbreviation defines the amplified CNV feature set and Del. abbreviation identifies genes in the deleted CNV feature set. The significantly identified KEGG pathways are presented for each feature class.
(TIF)

Figure S8 **Network for ovarian cancer identified tumor suppressors and oncogenes.** Genes with a strong correlation for methylation dependent expression exhibited at varying copy number aberrations identified in both the MSKCC and TCGA data sets include CDCA8, ATAD2, CDKN2A, and AURKA (blue circles). Here are depicted the functional relationships (regulating and binding) for those four genes with other known tumor suppressors and oncogenes.
(TIF)

Table S1 **MOMA identified hypomethylated and hypermethylated genes in ovarian cancer tumor samples for MSKCC data set.** Tumor to Normal methylation ratios are presented for hypomethylated and hypermethylated genes in 42 MSKCC samples from the MOMA platform. All MOMA data can be found in the GEO database (http://www.ncbi.nlm.nih.gov/geo/) for the subseries reference identifier GSE27940.
(XLS)

Table S2 **Correlation values per gene for copy number variation, methylation, and expression.** Correlation values for gene expression with copy number variation and separately methylation are presented for each gene in the **A**) MSKCC data set and **B**) TCGA data set. Significance values of Euclidean distances calculated between normal and tumor samples for methylation and expression data points for all genes in all samples and either in a deleted or amplified state is provided in the final three columns.
(XLS)

Table S3 **Ovarian cancer genes with large expression deviation within TCGA data set.** Genes with high and low CNV in at least 20% of TCGA data set samples are presented for expression deviations from normal.
(XLS)

Table S4 **All genes captured by Wilcoxon rank test based on copy number variation and expression data.** Results of Wilcoxon rank test with BH correction of selected genes in ovarian cancer tumor samples from both MSKCC and TCGA data sets. Bold indicate genes captured from both data sets.
(XLS)

Table S5 **Predicted genes with copy number variation, methylation and expression for tumor suppressor and oncogene features in both MSKCC and TCGA data sets.** Genes predicted from tumor suppressor and oncogene genomic and epigenetic features are shown. Location for each gene and epigenetic and genomic data values are shown. Thresholds used for each individual platform are provided per feature set.
(XLS)

Table S6 **Copy number derived methylation and expression of tumor suppressors and oncogenes.** Methylation and expression properties per copy number variation (from both the MSKCC and TCGA data sets) a presented for a curated list of known genes implicated in ovarian cancer or shown to be significant in cancer pathogenesis.
(XLS)

Analysis S1 **Supporting analysis scripts.**
(PDF)
