# Supplementary figures and images for: Identification of Tumor Suppressors and Oncogenes from Genomic and Epigenetic Features in Ovarian Cancer
Source: PLoS One. 2011 Dec 8;6(12):e28503. doi: 10.1371/journal.pone.0028503 (PMC3234280; doi:10.1371/journal.pone.0028503)

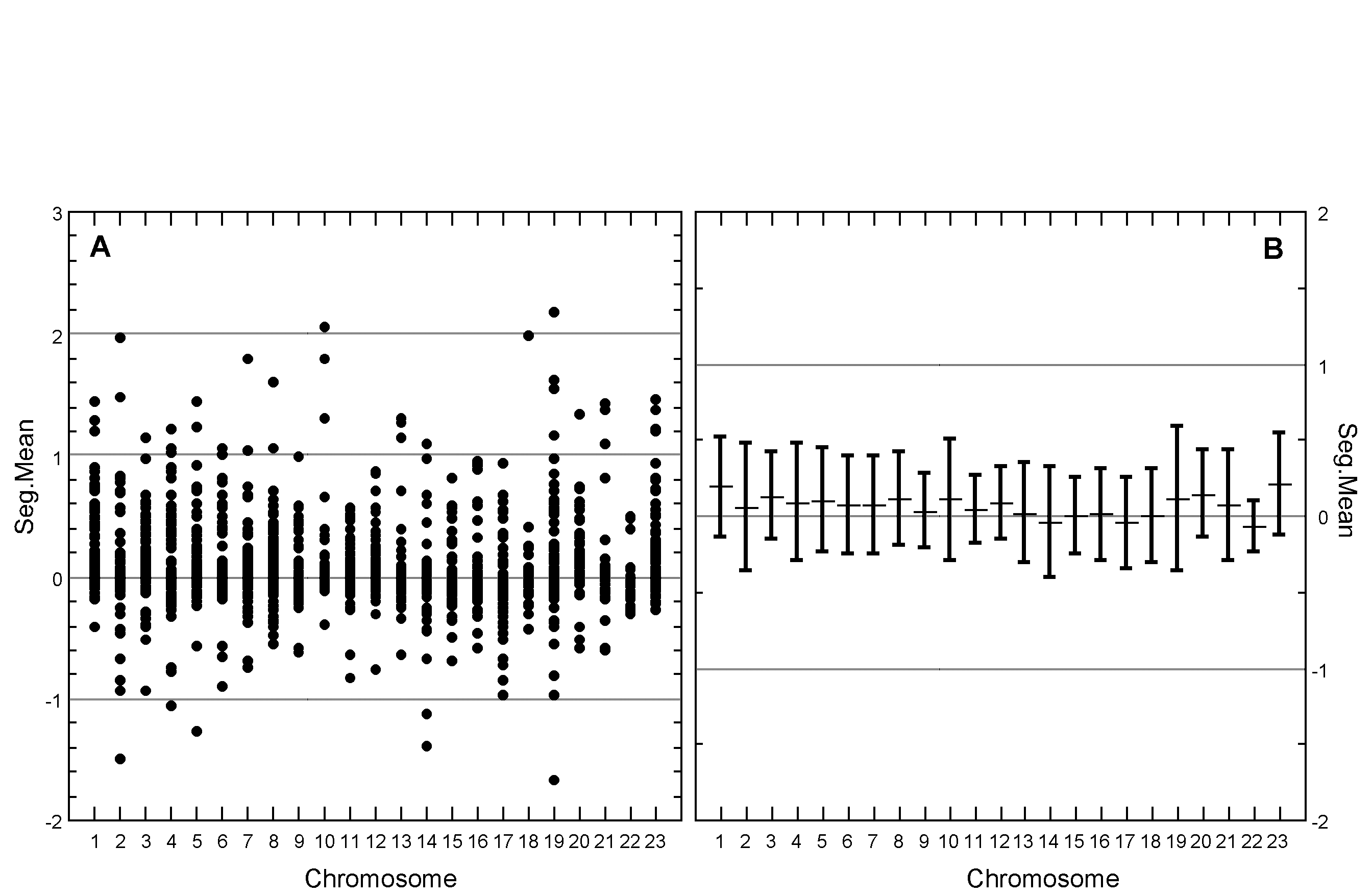

Supplement: Figure S1 — Copy number variability in ovarian cancer tumor samples. A) Variability per chromosome of all ROMA derived CBS segmentation values for 42 tumor samples in the MSKCC data set is shown. B) The mean value (horizontal straight bar) of CNV segmentation values per chromosome and standard deviation (error bars) from 42 tumor samples. (TIF) [file pone.0028503.s001.tif]

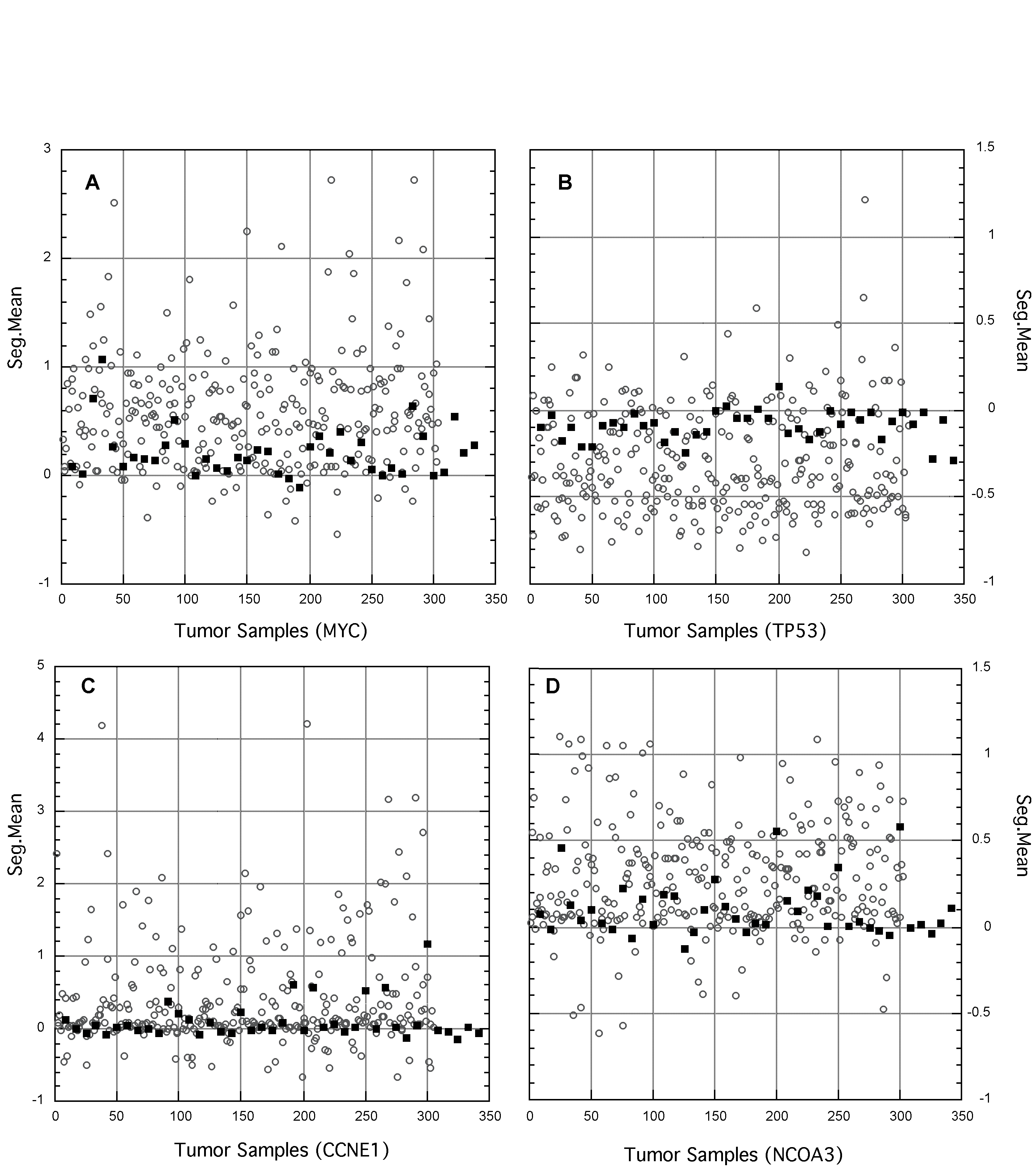

Supplement: Figure S2 — Copy number variation in TCGA and ROMA samples for ovarian cancer specific genes. Presented are four examples of copy number variation analyzed per gene (see methods) from TCGA tumor CNV data (open grey circles) and from MSKCC data set ROMA array tumor samples (filled black boxes). CBS segmentation mean (Seg.Mean) values per sample are plotted for four known ovarian cancer significant genes. Amplification and deletion sample comparisons between TCGA and ROMA segmentations are shown for A) MYC, B) TP53, C) CCNE1 and D) NCOA3. (TIF) [file pone.0028503.s002.tif]

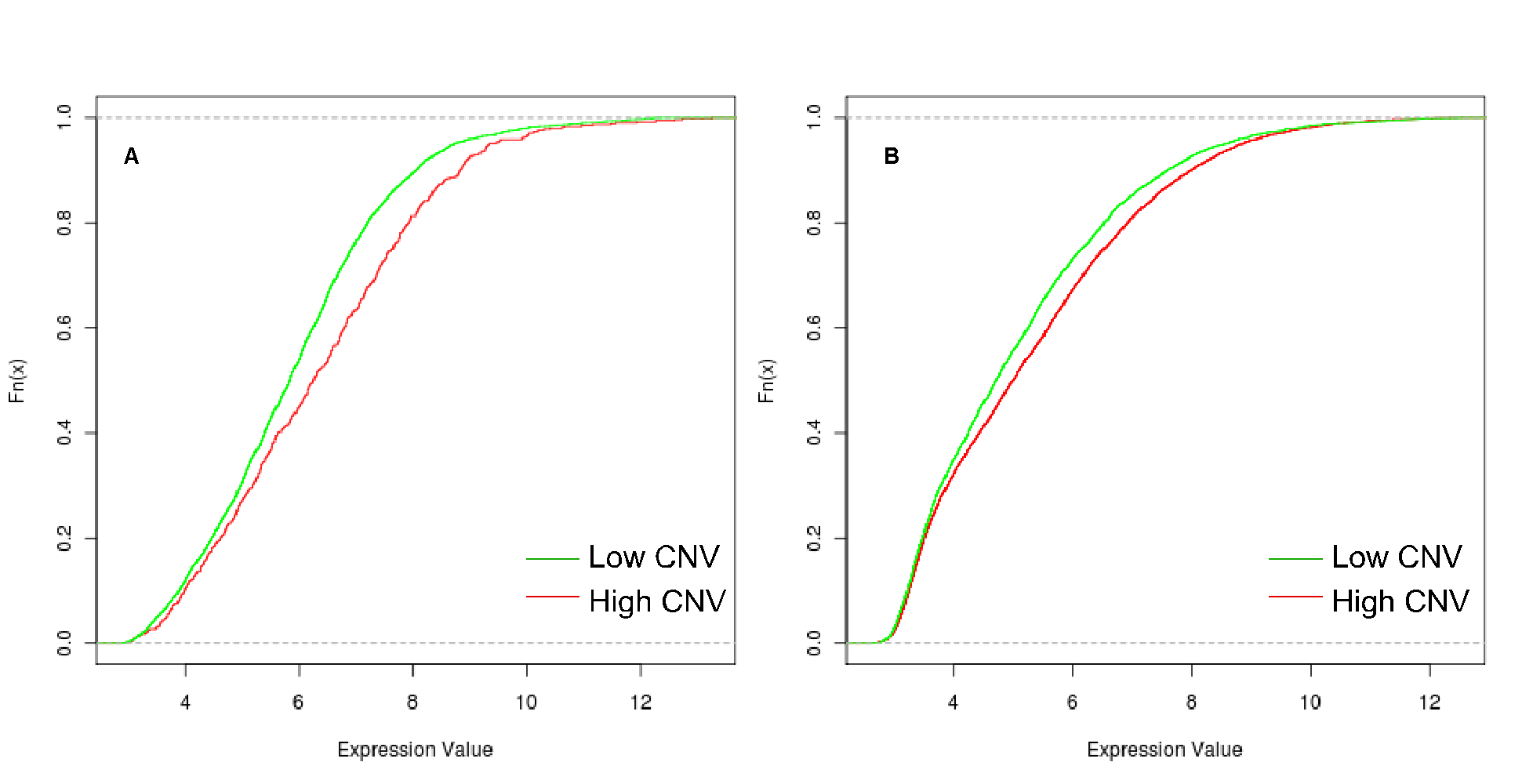

Supplement: Figure S3 — Cumulative distribution of gene expression per copy number variation. The cumulative distribution function (Fn(x) = P(X≤x)) for expression is plotted for genes with high (red line) and low (green line) copy number variation discovered from ROMA analysis (A) and found in the TCGA data set (B). Maximum difference in expression distribution between low and high copy is 7% in the TCGA data set and 17% in the MSKCC data set. (TIF) [file pone.0028503.s003.tif]

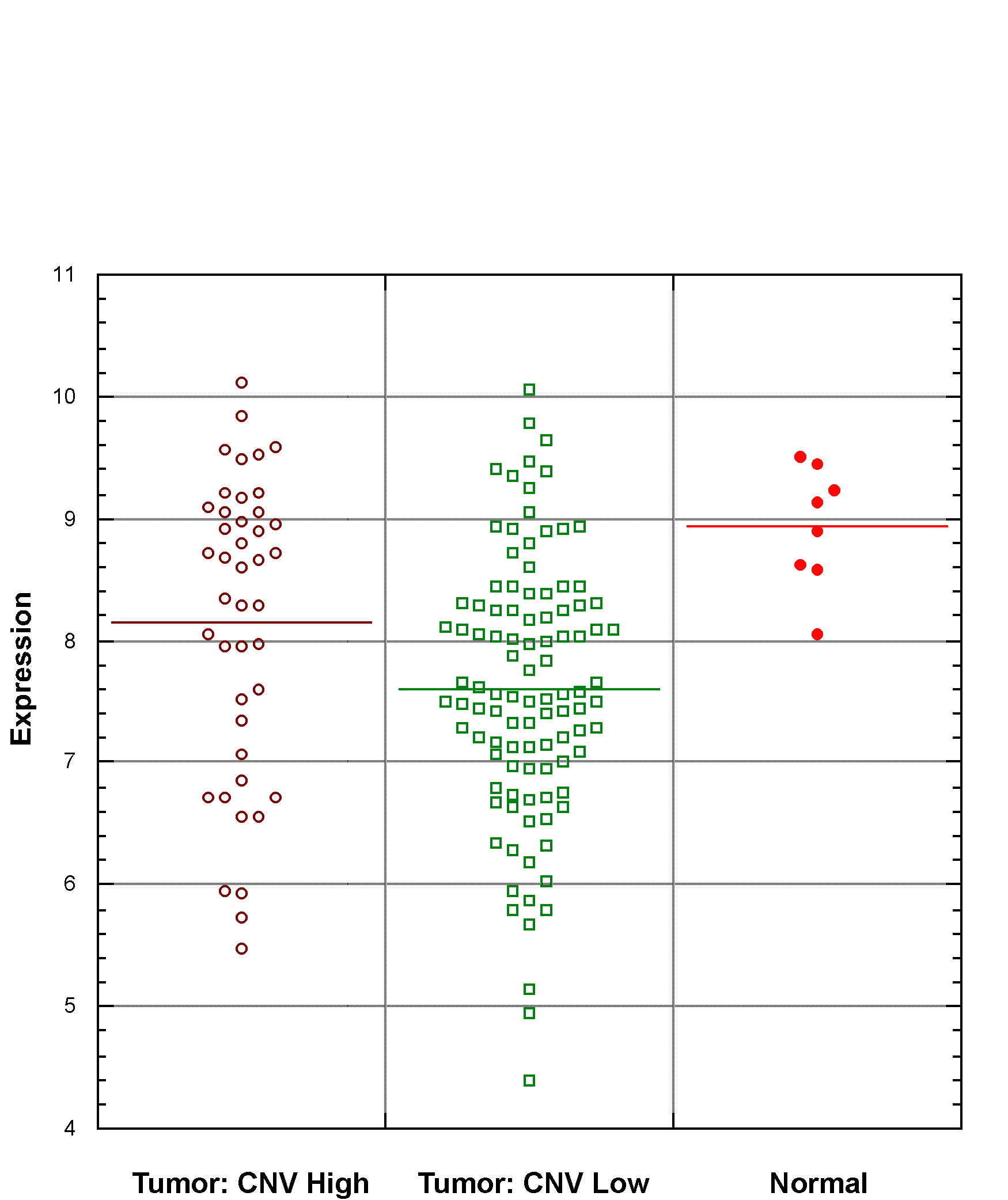

Supplement: Figure S4 — Expression of the MYC gene in ovarian tumor and normal samples in the TCGA data set. The expression of the gene MYC in ovarian tumor samples and normal samples as identified in the TCGA data set. Expression values are shown for samples with amplified CNV for MYC (left panel), deleted CNV for MYC (center panel) and for normal tissue (right panel). Colored bar shows the expression mean for each condition. (TIF) [file pone.0028503.s004.tif]

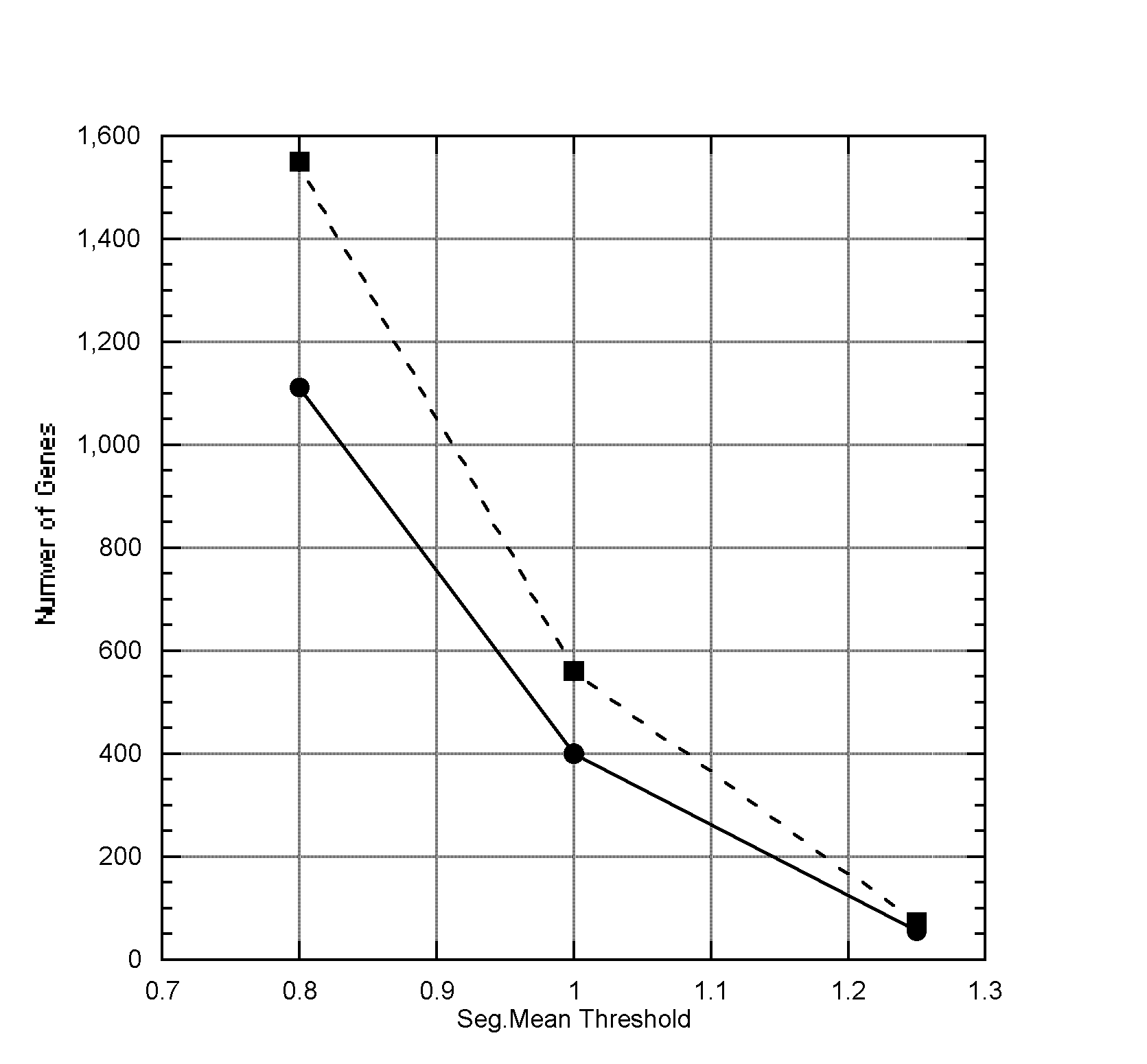

Supplement: Figure S5 — Genes captured by Wilcoxon rank test in ovarian cancer tumor samples. The Wilcoxon Rank test was performed on the ovarian cancer tumor TCGA data set. The test ranked expression levels of genes among samples with high and low copy number gene values. With a low CNV seg.mean threshold set at −0.50, the total genes captured was dependent on the high CNV threshold. Shown are the total genes captured (filled in square, dotted line) and number of genes with a FDR<0.50 (filled in circle, solid line) by the Wilcoxon rank test at CNV values of 0.80, 1.0 and 1.25. A total of 54 to 1114 genes with FDR<0.50 is identified using CNV threshold values of 0.80 to 1.25. (TIF) [file pone.0028503.s005.tif]

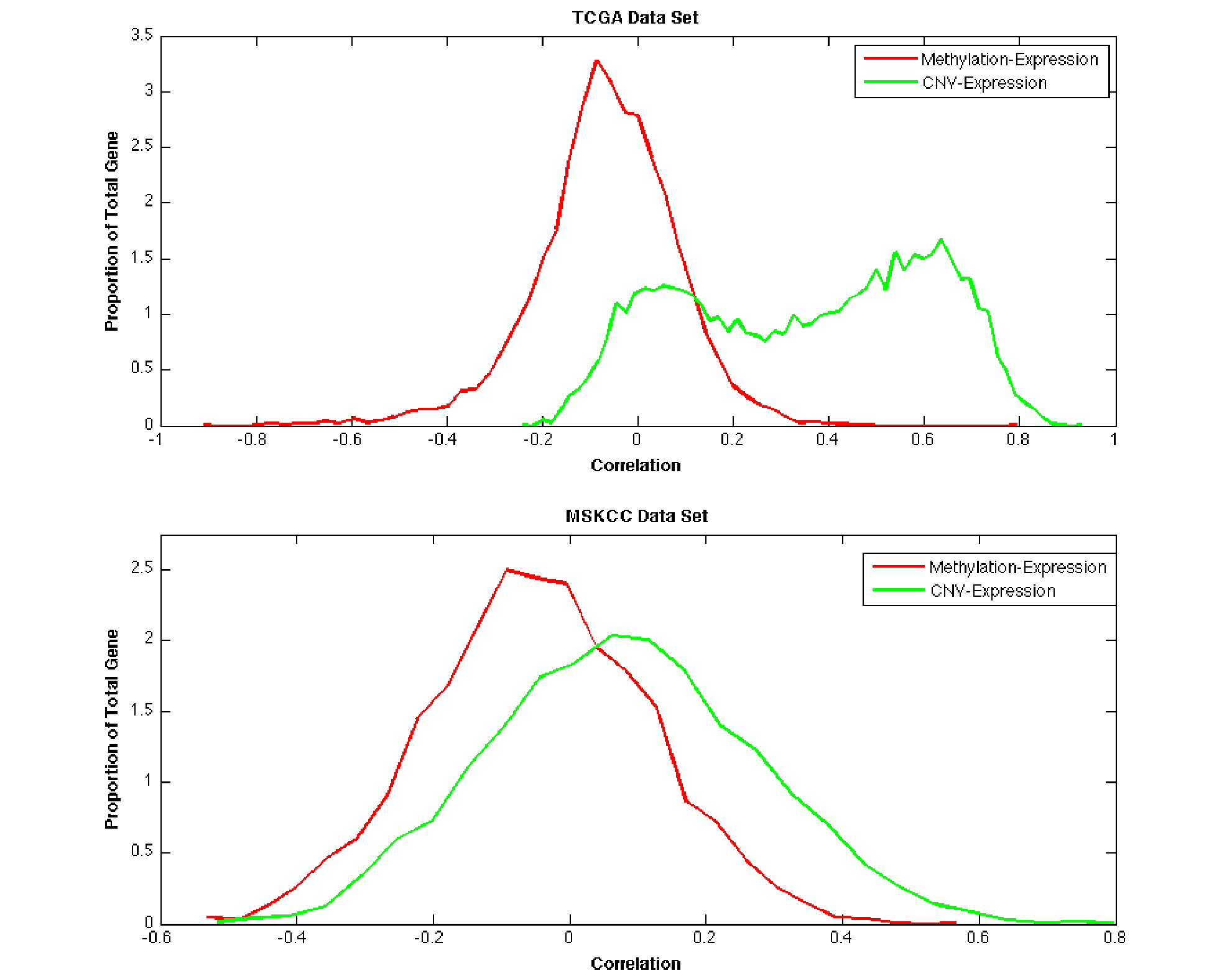

Supplement: Figure S6 — Expression correlation with copy number variation or methylation. Correlation value distribution per gene of expression to copy number variation (CNV-Expression, green line) and methylation (Methylation-Expression, red line) are shown as a proportion of total genes analyzed for TCGA (A) and ROMA-MOMA MSKCC data (B). (TIF) [file pone.0028503.s006.tif]

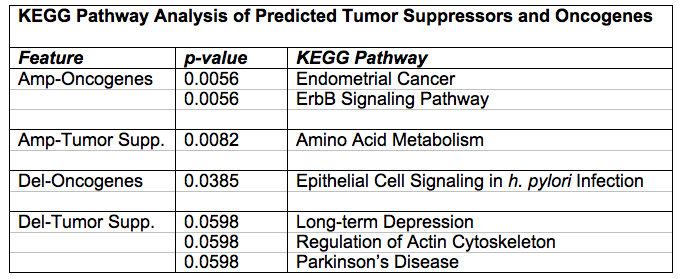

Supplement: Figure S7 — KEGG pathway enrichment analysis. A KEGG pathway enrichment analysis defined by hypergeometric distribution was performed on the genes predicted in the MSKCC data set for each genomic and epigenetic feature class for oncogenes and tumor suppressors. Amp. abbreviation defines the amplified CNV feature set and Del. abbreviation identifies genes in the deleted CNV feature set. The significantly identified KEGG pathways are presented for each feature class. (TIF) [file pone.0028503.s007.tif]

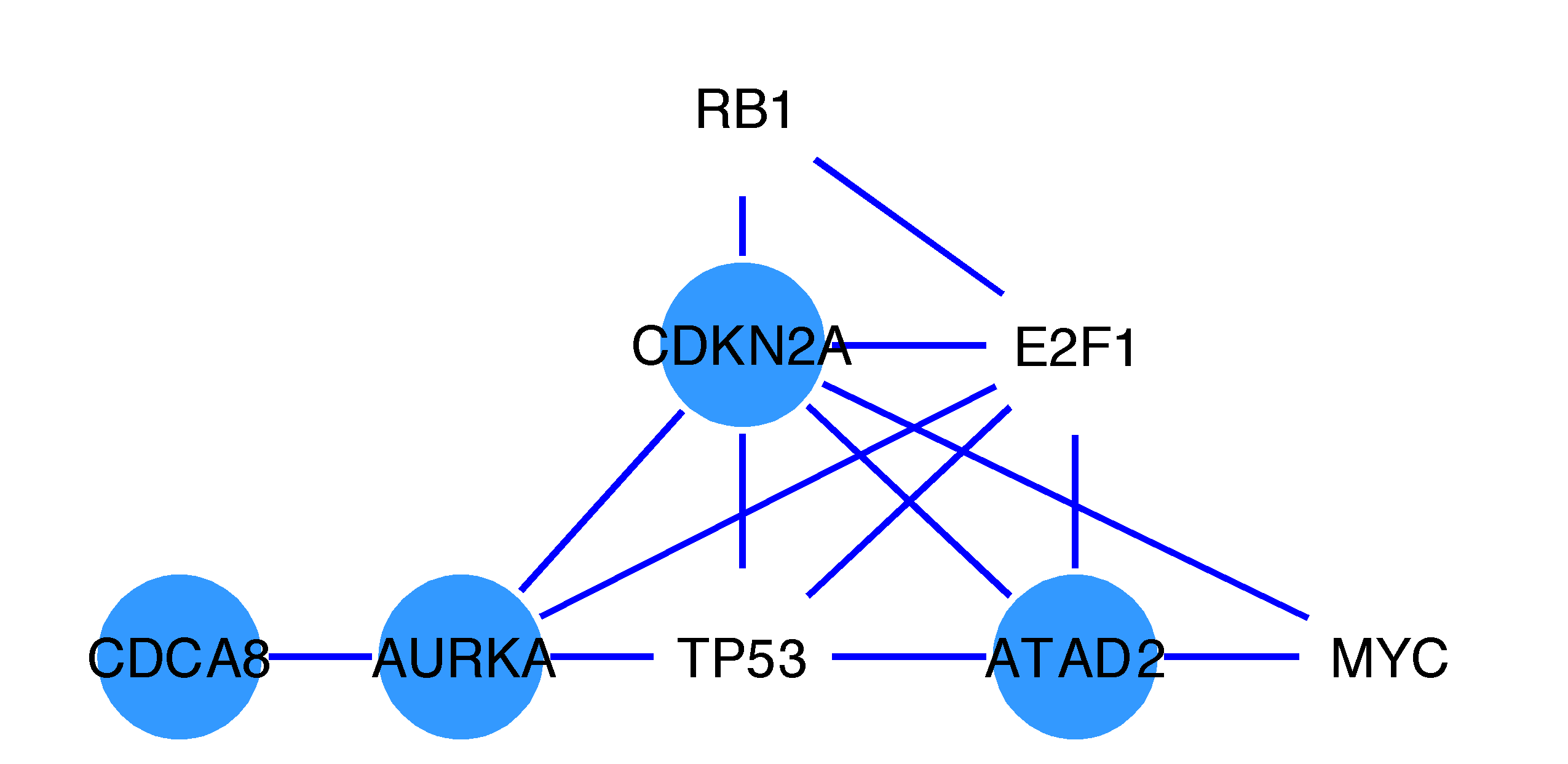

Supplement: Figure S8 — Network for ovarian cancer identified tumor suppressors and oncogenes. Genes with a strong correlation for methylation dependent expression exhibited at varying copy number aberrations identified in both the MSKCC and TCGA data sets include CDCA8, ATAD2, CDKN2A, and AURKA (blue circles). Here are depicted the functional relationships (regulating and binding) for those four genes with other known tumor suppressors and oncogenes. (TIF) [file pone.0028503.s008.tif]
