# Supplementary material for: Identification of Tumor Suppressors and Oncogenes from Genomic and Epigenetic Features in Ovarian Cancer
Source: PLoS One. 2011 Dec 8;6(12):e28503. doi: 10.1371/journal.pone.0028503 (PMC3234280; doi:10.1371/journal.pone.0028503)
Supplement: Analysis S1 — Supporting analysis scripts. (PDF) [file pone.0028503.s015.pdf]

Supplementary scripts for PLoS One Manuscript:
"Identification of Tumor Suppressors and Oncogenes from Genomic and Epigenetic Features in Ovarian Cancer"
Kazimierz O. Wrzeszczynski, Vinay Varadan, James Byrnes, Elena Lum, Sitharthan Kamalakaran, Douglas A. Levine, Nevenka Dimitrova, Michael Q. Zhang, Robert Lucito.

contact information:
Kazimierz O. Wrzeszczynski Ph.D.
Cold Spring Harbor Laboratory
Bioinformatics and Genomics
Cold Spring Harbor, NY 11724.
e-mail: kwrzeszc@cshl.edu
tel. 516-367-8395

All raw and normalized data is publically available on the NCBI GEO site: Series GSE28015, GSE28013, GSE27943, GSE27940.
Initial experimental data compilation was performed with preliminary scripts and packages publicly available via S-plus or R Bioconductor.
Matlab was used to make figures. Scripts in this Supplementary file are final scripts used for all figures and data analysis in the manuscript and can be incorporated with other CNV, Methylation and Expression data.
For publication guidelines and submission ease all scripts are reprinted into this .pdf file.
All data analysis scripts, raw data files, and further assistance or information regarding data analysis can be obtained directly by contacting: kwrzeszc@cshl.edu

```
###### - kegg_pathway_enrichment.r ###########
#Vinay Vardan, K.O. Wrzeszczynski
#Philips Research North America, Cold Spring Harbor Laboratory.
#copyright 2011.
#Kegg Pathway Enrichment Analysis.
#must download required files from KEGG.
#gene input files are single column list of genes.
#email contact: kwrzesz@cshl.edu

require(KEGG.db)
require(hgu95av2.db)
require(multtest)
require(KEGG.db)
setwd('[/home/working/directory]') #Set to where pathway ids file is stored.
pathway_ids = read.table('hsa_pathway_ids.txt',sep='\t', header=F) #must download pathway id's
 from KEGG.
pathway_ids = as.character(as.matrix(unlist(pathway_ids))
totalgenes = read.table('allgenes.txt', header=F); #all identified genes.
predgenes = read.table('featuregene.txt', header=F) #feature class predicted gene set.


totalgenes=as.matrix(totalgenes)
predgenes=as.matrix(predgenes)
library(hgu95av2.db)
library(multtest)
library(KEGG.db)
library(org.Hs.eg.db)

find_hyp_pval <- function(a)
{
  pathgeneids = get(a, KEGGPATHID2EXTID)
  pathgenenames = as.matrix(unlist(mget(pathgeneids, org.Hs.egSYMBOL)))
  pathwaygenesintotalgenes = intersect(pathgenenames, totalgenes) #genes in KEGG pathway
 that are in my total gene list
  number_of_pathwaygenesintotalgenes = length(pathwaygenesintotalgenes)
  pathwaygenesinPred = intersect(predgenes, pathgenenames) #gives genes from the redicted gene
 set that are in the pathway
  number_pathwaygenesinPred = length(pathwaygenesinPred)
  number_totalgenes = length(totalgenes) #number of genes you started with
  num_Predgenes = length(predgenes)
  pval = phyper(number_pathwaygenesinPred, number_of_pathwaygenesintotalgenes,
 number_totalgenes-number_of_pathwaygenesintotalgenes, num_Predgenes, lower.tail = FALSE,
 log.p = FALSE)
  out <- c(a, pval, number_pathwaygenesinPred, number_of_pathwaygenesintotalgenes,
 length(pathgeneids))
}
temp<-as.matrix(pathway_ids)
k<-apply(temp,1,find_hyp_pval)
results_for_all_pathways<-t(k)
tmpsor =  sort(as.numeric(results_for_all_pathways[,2]), decreasing = FALSE, index.return =
```

```
 TRUE)
k2 = results_for_all_pathways[tmpsor$ix,]
temp1<-mt.rawp2adjp(as.numeric(k2[,2]),proc="BH")
k2 = cbind(k2[,1:2], as.matrix(temp1$adjp[,2]), k2[,3:5])
pathnames = read.delim('hsa_pathways.txt', sep="\t", header=F)
inds = match(k2[,1], pathnames[,1])
final_results_all_pathways = cbind(k2, as.vector(pathnames[inds,3]))
colnames(final_results_all_pathways) = c('PathwayID', 'Pvalue', 'BH',
 'NumberPathwayGenesinPred', 'NumberPathwayGenesinTotal','NumberGenesinPathway',
 'Pathway Name')
write.table(final_results_all_pathways, file="final_results_AMP-TS_pathways.txt", col.names =
 TRUE, row.names=FALSE)
```

```
####### MSKCC_CNV_EXP_ME_sample_frequency_per_gene.pl ############
#! /user/bin/perl -w
##################################################################################
#Kazimierz O. Wrzeszczynski Ph.D.
#Cold Spring Harbor Laboratory, Cold Spring Harbor, NY 11724
#copyright 2011
#takes each sample and sorts the genes by 3 stages of CNV then calculates
#the average and median epigentic data.
#input file is tab delimited data for each sample, 7 columns with format:
##Chromosome   TSS.pos Sample  Gene    CNV     ExpVal  MetVal
##################################################################################


$datafile= " [ input file with per sample gene CNV, Expression and Methylation data ] " ;

open (FHFILE, $datafile) or die "could not open $datafile \n";
while (<FHFILE>){
   if ($_=~ /^\s|\#|Chromosome/){next;}

   $row=$_;
   chomp($row);
   @line=splt(/\t/,$row);

   $chr=$line[0];
   $pos=$line[1];
   $sid=$line[2];
   $gene=$line[3];
   $cnv=$line[4];
   $exp=$line[5];
   $met=$line[6];
   $metdata{$gene}{$sid}=$met;
   $expdata{$gene}{$sid}=$exp;
   $cnvdata{$gene}{$sid}=$cnv;
   $genep{$gene}=$gene;

   }
}
close FHFILE;


foreach $gid (keys %genep){
 @Cmevalues=();
 @Bmevalues=();
 @Amevalues=();
 @Cexpvalues=();
 @Bexpvalues=();
 @Aexpvalues=();
 @Ccnvvalues=();
 @Bcnvvalues=();
 @Acnvvalues=();

   $totalAme=0;
   $totalAcnv=0;
   $totalAexp=0;
   $totalBme=0;
```

```
    $totalBcnv=0;
    $totalBexp=0;
    $totalCme=0;
    $totalCcnv=0;
    $totalCexp=0;
    $totalme=0;
    $totalcnv=0;
    $totalexp=0;

    $cnt=0;

    $avgAme{$gid}=0;
    $avgAcnv{$gid}=0;
    $avgAexp{$gid}=0;
    $avgBme{$gid}=0;
    $avgBcnv{$gid}=0;
    $avgBexp{$gid}=0;
    $avgCme{$gid}=0;
    $avgCcnv{$gid}=0;
    $avgCexp{$gid}=0;

    $poscnv=0;
    $negcnv=0;
    $medcnv=0;
    $tcnv=0;



    foreach $sid (keys %{$metdata{$gid}}) {
            $cnt++;
            $totalme += $metdata{$gid}{$sid};
            $totalcnv += $cnvdata{$gid}{$sid};
            $totalexp += $expdata{$gid}{$sid};

            $cnvampthresh = 0.50; #Amp threshold variable
        $cnvdelthresh = -0.50; #Del threshold variable
            if ($cnvdata{$gid}{$sid} >= $cnvampthresh ){$poscnv++;
                                    push(@Acnvvalues, $cnvdata{$gid}{$sid});
                                    push(@Aexpvalues, $expdata{$gid}{$sid});
                                    push(@Amevalues, $metdata{$gid}{$sid});
                                    $totalAme += $metdata{$gid}{$sid};
                                    $totalAexp += $expdata{$gid}{$sid};
                                    $totalAcnv += $cnvdata{$gid}{$sid};
            }
        if  (($cnvdata{$gid}{$sid}  <  $cnvampthresh  )  &&  ($cnvdata{$gid}{$sid}  >  $cnvdelthresh)
){$medcnv++;
                                    push(@Bcnvvalues, $cnvdata{$gid}{$sid});
                                    push(@Bexpvalues, $expdata{$gid}{$sid});
                                    push(@Bmevalues, $metdata{$gid}{$sid});
                                    $totalBme += $metdata{$gid}{$sid};
                                    $totalBexp += $expdata{$gid}{$sid};
                                    $totalBcnv += $cnvdata{$gid}{$sid};
            }


            if ($cnvdata{$gid}{$sid} <= $cnvdelthresh){ $negcnv++;
```

```
                                        push(@Ccnvvalues, $cnvdata{$gid}{$sid});
                                        push(@Cexpvalues, $expdata{$gid}{$sid});
                                        push(@Cmevalues, $metdata{$gid}{$sid});
                                        $totalCme += $metdata{$gid}{$sid};
                                        $totalCexp += $expdata{$gid}{$sid};
                                        $totalCcnv += $cnvdata{$gid}{$sid};
          }

  }



  $tcnv=$poscnv+$negcnv+$medcnv;




 if ($poscnv ==0 ){
    $avgAme{$gid}=0;
    $avgAexp{$gid}=0;
    $avgAcnv{$gid}=0;
}
else {
    $avgAme{$gid}=$totalAme/$poscnv;
    $avgAexp{$gid}=$totalAexp/$poscnv;
    $avgAcnv{$gid}=$totalAcnv/$poscnv;
    $stdcnvA{$gid}= stdev(\@Acnvvalues,  $avgAcnv{$gid});
    $stdmeA{$gid}= stdev(\@Amevalues,  $avgAme{$gid});
    $stdexpA{$gid}= stdev(\@Aexpvalues,  $avgAexp{$gid});
}


if ($medcnv ==0 ){
    $avgBme{$gid}=0;
    $avgBexp{$gid}=0;
    $avgBcnv{$gid}=0;
}
else {

    $avgBme{$gid}=$totalBme/$medcnv;
    $avgBcnv{$gid}=$totalBcnv/$medcnv;
    $avgBexp{$gid}=$totalBexp/$medcnv;
    $stdcnvB{$gid}= stdev(\@Bcnvvalues,  $avgBcnv{$gid});
    $stdmeB{$gid}= stdev(\@Bmevalues,  $avgBme{$gid});
    $stdexpB{$gid}= stdev(\@Bexpvalues,  $avgBexp{$gid});
}



 if ($negcnv ==0 ){
    $avgCme{$gid}=0;
    $avgCexp{$gid}=0;
    $avgCcnv{$gid}=0;
}
else {
```

```
    $avgCexp{$gid}=$totalCexp/$negcnv;
    $avgCcnv{$gid}=$totalCcnv/$negcnv;
    $avgCme{$gid}=$totalCme/$negcnv;

    $stdcnvC{$gid}= stdev(\@Ccnvvalues,  $avgCcnv{$gid});
    $stdexpC{$gid}= stdev(\@Cexpvalues,  $avgCexp{$gid});
    $stdmeC{$gid}= stdev(\@Cmevalues,  $avgCme{$gid});
}

    $countsup{$gid} = $poscnv;
    $countsmed{$gid} = $medcnv;
    $countsneg{$gid} = $negcnv;

    $medcnvB{$gid}= median(\@Bcnvvalues);
    $medcnvC{$gid}= median(\@Ccnvvalues);
    $medcnvA{$gid}= median(\@Acnvvalues);

    $medexpB{$gid}= median(\@Bexpvalues);
    $medexpC{$gid}= median(\@Cexpvalues);
    $medexpA{$gid}= median(\@Aexpvalues);

    $medmeB{$gid}= median(\@Bmevalues);
    $medmeC{$gid}= median(\@Cmevalues);
    $medmeA{$gid}= median(\@Amevalues);

}



print "CNV\tcounts\taCNV\tmCNV\taMe\tmMe\taExp\tmExp\n";

#For a secific set of "your favorite genes" array, can also be inputed as separate list file with
@yfg=(<$ARGV[0]>);

@yfg = ("ARID1A", "PPP2R1A", "SPARC", "KRAS", "MYC", "PRKC1", "CCNE1", "NKX2", "RAB25",
"AURKA", "CDKN2A", "PIK3CA", "PIK3R1", "DAB2", "CASP9", "PTEN", "TMPRSS2", "ERG",
"ERBB2", "RPS6KA2", "NOTCH3", "ETV6", "RB1", "TP53", "PLAGL1", "WWOX", "EGFR",
"BRCA1", "BRCA2", "DIRAS3", "PEG3", "FGFR1", "FGF1", "EIF5A2", "DLEC1", "AKT2", "OPCML",
"WFDC2", "MUC16", "MLH1", "ITGB1", "CD44", "MDM2", "MDM4",
"IDH1","IDH2","IDH3A","IDH3B","IDH3G", "SMARCA4", "BRG1", "KDM5C", "JARID1C", "GNAQ",
"ID4", "PEA15", "GAGE4", "GAGE6", "CALR", "CD47", "JAK2", "SIRPA", "MLL", "MLL4",
"SMARCA4", "EED", "SMARCD1", "SUZ12", "MEN1", "DUB3", "CDC25A", "PIAS3", "STAT3",
"ABL1", "PDGFRA", "NF1", "CDK4", "ATAD2", "CDCA8", "BOP1", "EIF2C3", "E2F1", "EIF2A1",
"MLL", "AMD1", "FBXW7", "MECOM", "ZMYND8", "ID4", "TERT");

  foreach $gid (@yfg) {  #(keys %genep){ #used for all genes in data file.

    $total=0;
        $frequp=0;
        $freqmed=0;
    $freqneg=0;

        $total= $countsup{$gid} + $countsmed{$gid} + $countsneg{$gid};

        if ($total == 0){
                $frequp=0;
```

```
		$freqmed=0;
			$freqneg=0;}
		else {
		$frequp = $countsup{$gid}/$total;
		$freqmed = $countsmed{$gid}/$total;
		$freqneg = $countsneg{$gid}/$total;
		}

		printf	("$gid\tAmplified\t%2.2f\t%2.2f\t%2.2f\t%2.2f\n",	$frequp,	$avgAcnv{$gid},
$avgAme{$gid}, $avgAexp{$gid});
		printf	("$gid\tNeutral\t%2.2f\t%2.2f\t%2.2f\t%2.2f\n",	$freqmed,	$avgBcnv{$gid},
$avgBme{$gid}, $avgBexp{$gid});
		printf	("$gid\tDeleted\t%2.2f\t%2.2f\t%2.2f\t%2.2f\n",	$freqneg,	$avgCcnv{$gid},
$avgCme{$gid}, $avgCexp{$gid});

}

##############Subroutines######################################
## subroutines only accept scalar arguments. change @ to scalar above with \@

sub median {
#@_ == 1 or die ('Sub usage: $median = median(\@array);');
my ($array_ref) = @_;
my $count = scalar @$array_ref;
#$count = $#array;
#Sort a COPY of the array, leaving the original untouched
my @array = sort { $a <=> $b } @$array_ref;
if ($count % 2) {
return $array[int($count/2)];
} else {
#return ($array[$count/2] + $array[$count/2 - 1]) / 2; #takes average or two middle
return ($array[$count/2 -0.5])
}
}


sub stdev {
   #my @arr_ref=();
   #my $avg=0;

   ($arr_ref, $avg)=@_;

   my $count=0;
   $count = scalar @$arr_ref;
   #print "count - $count\t $avg\n";

  $x=0; $xsq=0; $sumx=0; $d=0;
   $sd=0;


   if ($count >1){
   foreach $point (@$arr_ref){
   $x = $point-$avg;
   $xsq=$x*$x;
   $sumx += $xsq;
   $d=$sumx/($count-1);
```

```
    $sd = sqrt($d);
    $sd=sprintf("%2.4f", $sd);
    }
    }
    else {$sd=0.00;}

    return ($sd);
}
#end of script.
```

```
############## ROMA_EXP_MOMA_freq_pergene_TNratio.pl ##################
###########################################################
#   Kazimierz O. Wrzeszczynski Ph.D.
#   CSHL, Cold Spring Harbor, NY 11724
#   copyright 2011.
#
#   perl script to parse out TS/ONC Feature genes
#
###########################################################

#! /user/bin/perl -w

use Time::localtime;
$datafile= #[datafile.txt] #contains cnv, ex and methyaltion data;
#Chromosome     TSS.pos Sample  Gene     CNV     ExVal   MOMA
#1        11866291650.JB0172      TNFRSF4          0.5947  3.99      1.058
$normalex= # [t/n expression file ] #Tumor/Normal Expression Ratio file. Format is Gene and Expression Ratio.

open (FHFILENEX, $normalex) or die "could not open $normalex\n";
while (<FHFILENEX>){

if ($_=~ /^\s|\#|Chromosome/){next;}
$row=$_;
chomp($row);
@linea = split(/\t/, $row);

$gene=$linea[0];
$nex=$linea[1];

$norm{$gene}=$nex;

}
close FHFILENEX;

open (FHFILE, $datafile) or die "could not open $datafile \n";
while (<FHFILE>){

if ($_=~ /^\s|\#|Chromosome/){next;}


$row=$_;
chomp($row);
@linea = split(/\t/, $row);

$sid=$linea[2];
$chr=$linea[0];
$start=$linea[1];
$gene=$linea[3];
$cnv=$linea[4];
$exp=$linea[5];
$met=$linea[6];

$metdata{$gene}{$sid}=$met;
$expdata{$gene}{$sid}=$exp;
```

```
$cnvdata{$gene}{$sid}=$cnv;
$genepos{$chr}{$gene}=$start;

}
close FHFILE;

foreach $gid (keys %metdata){

    $totalme=0;
    $totalcnv=0;
    $totalexp=0;
    $totalhighexp=0;
    $totallowexp=0;
    $totallowmet=0;
    $totalhighmet=0;
    $avghighexp=0;
    $avglowexp=0;
    $avglowmet=0;
    $avghighmet=0;
    $cnt=0;
    $avgme=0;
    $avgcnv=0;
    $avgexp=0;
    $freqme=0;
    $freqcnv=0;
    $freqexp=0;
    $posme=0;
    $negme=0;
    $poscnv=0;
    $negcnv=0;
    $posexp=0;
    $negexp=0;
    $texp=0;
    $tme=0;
    $tcnv=0;

    foreach $sid (keys %{$metdata{$gid}}) {
                $hypermet = #set hypermthylation threshold.
                $hypomet = # set hypomethylation threshold.
                $cnvrangea = #set upper CNV value.
                $cnvrangeb = #set lower CNV value.
                $expa = #set upper expression value.
                $expb = #set lower expression value.
                $cnt++;
                $totalme += $metdata{$gid}{$sid};
                $totalcnv += $cnvdata{$gid}{$sid};
                $totalexp += $expdata{$gid}{$sid};
          if ($metdata{$gid}{$sid} > $hypermet){$posme++;}
                if ($metdata{$gid}{$sid} <= $hypomet ){$negme++;}

               #LOW: change to nfrequencycnv and lowcnvexp in printout line below
                if ($cnvdata{$gid}{$sid} >    $cnvrangea){$poscnv++; $totalhighexp+=$expdata{$gid}{$sid};
}
                if ($cnvdata{$gid}{$sid} <=   $cnvrangeb){$negcnv++; $totallowexp+=$expdata{$gid}{$sid};
$totallowmet+=$metdata{$gid}{$sid};     }
```

```
        #HIGH: change to frequencycnv and highcnvexp in printout line below

            #   if ($cnvdata{$gid}{$sid} >= $cnvrangea){$poscnv++; $totalhighexp+=$expdata{$gid}{$sid};
$totalhighmet+=$metdata{$gid}{$sid};   }
            #    if ($cnvdata{$gid}{$sid} < $cnvrangeb){$negcnv++; $totallowexp+=$expdata{$gid}{$sid};
}


            if ($expdata{$gid}{$sid} >= $expa){$posexp++;}
            if ($expdata{$gid}{$sid} < $expb ){$negexp++;}

    }

    $avgme=$totalme/$cnt;
    $avgcnv=$totalcnv/$cnt;
    $avgexp=$totalexp/$cnt;

    if (($poscnv == 0) || ($totalhighexp==0)){
            $highcnvexp{$gid}=0;
    }
    if (($poscnv != 0) && ($totalhighexp != 0)){
    $avghighexp=$totalhighexp/$poscnv;
    $highcnvexp{$gid}=$avghighexp;
    $avghighmet=$totalhighmet/$poscnv;
    $highcnvmet{$gid}=$avghighmet;
}

    if (($negcnv == 0) || ($totallowexp==0)){
            $lowcnvexp{$gid}=0;
    }
    if (($negcnv != 0) && ($totallowexp != 0)){
    $avglowexp=$totallowexp/$negcnv;
    $lowcnvexp{$gid}=$avglowexp;
    $avglowmet=$totallowmet/$negcnv;
    $lowcnvmet{$gid}=$avglowmet;
}



    $texp=$posexp+$negexp;
    $tcnv=$poscnv+$negcnv;
    $tme=$posme+$negme;

    $freqexp=$posexp/$texp;
    $freqcnv=$poscnv/$tcnv;
    $nfreqcnv=$negcnv/$tcnv;
    $freqme=$posme/$tme;
    $finalme{$gid}=$avgme;
    $finalcnv{$gid}=$avgcnv;
    $finalexp{$gid}=$avgexp;
    $frequencyme{$gid}=$freqme;
    $frequencyexp{$gid}=$freqexp;
    $nfrequencycnv{$gid}=$nfreqcnv;
    $frequencycnv{$gid}=$freqcnv;
```

```
}

@hgid=();
@lgid=();



foreach $chr (sort {$a <=> $b} keys %genepos){

   foreach $gid (sort { $genepos{$chr}{$a} <=> $genepos{$chr}{$b} } keys %{$genepos{$chr}}){

            if ($nfrequencycnv{$gid} >= 0.10){ #frequency threshold
                        if (exists $norm{$gid}){
               #$ratio=$highcnvexp{$gid}/$norm{$gid};
               $ratio=$lowcnvexp{$gid}/$norm{$gid};


       #LOW
        printf   ("$gid\t$chr\t%9g\t%2.2f\t%2.4f\t%2.4f\n",   $genepos{$chr}{$gid},   $nfrequencycnv{$gid},
$ratio, $lowcnvmet{$gid});

       #High
       #printf   ("$gid\t$chr\t%9g\t%2.2f\t%2.4f\t%2.4f\n",   $genepos{$chr}{$gid},   $frequencycnv{$gid},
$ratio, $highcnvmet{$gid});


                        }
            }

   }
} #end foreach chr.
#end of script
```

```
#############  ROMA_variability.pl  #################

########################################################
#   Kazimierz O. Wrzeszczynski Ph.D.
#   CSHL - Cold Spring Harbor, NY 11724
#   copyright 2011.                              #
#   Script to identify variable regions in ROMA data
#   input is matrix of probe values per sample
########################################################
#! /user/bin/perl -w

$file=$ARGV[0]; # input probe first column sample and ROMA value in subsequent columns.

for ($g=2;$g<=42;$g++){
$data[$g]=0;
}

open (FHFILE, $file) or die "could not open $file\n";

while (<FHFILE>) {

 if ($_ =~ m/^CHROM/){

            $row=$_;
            chomp($row);
            @sample=split(/\t/, $row);
 }

   if ($_ =~ m/^\d+\s+\S+/){

            $row=$_;
            chomp($row);
            @line=split(/\t/, $row);
            $chr=$line[0];
            $bp=$line[1] * 1000000;

            for ($g=2;$g<=42;$g++){


               if ($line[$g] != $data[$g]){

               $cnt{$chr}{$g}++;
               $loci{$g}{$chr}{$cnt{$chr}{$g}}=$bp;
               $data[$g]=$line[$g];
               $roma{$g}{$chr}{$cnt{$chr}{$g}}=$data[$g];


               }
            }
   }
}
close FHFILE;


for ($g=2;$g<=42;$g++){
```

```
    for ($chr=1;$chr<=24;$chr++){

            for ($x=1;$x<=$cnt{$chr}{$g};$x++){


                $chro{$chr}{$g}{$x}=$roma{$g}{$chr}{$x};

            }
    }
}

for ($v=1;$v<=22;$v++){  #omit X and Y chromosome X is from reference.

   for ($g=2;$g<=42;$g++){
      for ($i=1;$i<=$cnt{$v}{$g};$i++){

    $pos{$v}{$loci{$g}{$v}{$i}}++;
    $possm{$v}{$loci{$g}{$v}{$i}}+=$chro{$v}{$g}{$i};
    $chkbos{$v}{$loci{$g}{$v}{$i}}=$loci{$g}{$v}{$i};


      }
   }
}




$totalbp=0; $prevbp=0; $addbp=0; $addsamps=0; $prevtot=0;

for ($v=1;$v<=22;$v++){
    $prevbp=0; $addbp=0;  $addsamps=0; $r=0; $avgavg=0; $j=0;
    LINE: foreach $locbp (sort {$chkbos{$v}{$a} <=> $chkbos{$v}{$b} } keys %{$chkbos{$v}}){
            $avg=$possm{$v}{$locbp}/$pos{$v}{$locbp};
            $addbp   = $locbp-$prevbp;
        $totalbp += $addbp;

            #print "$v\t$locbp\t$totalbp\t$pos{$v}{$locbp}\t$possm{$v}{$locbp}\t$avg\n";


            if ($pos{$v}{$locbp}  == 41)  {printf ("$v\t$locbp\t$totalbp\t$pos{$v}{$locbp}\t%2.4f\n", $avg);
next LINE;
                            }
        if ($addbp > 1000000){

                                    print "$v\t$locbp\t$totalbp\t$pos{$v}{$locbp}\t$avg\n";
                                    $j=0;  $r=0;
            }
            $prevbp=$locbp;
            $prevtot=$totalbp;
        if ($addbp <= 1000000) { $r++;
                                        $j += $pos{$v}{$locbp};
                                        $addsamps += $avg;
                                        $avgavg=$addsamps/$r;
                              print "$v\t$locbp\t$totalbp\t$pos{$v}{$locbp}\t$avg\n";
```

```
                    $j=0;  $r=0;    }
          }

    }
#end of script
```

```
####### wilcox_r_wrapper_for_ROMA.pl ################
###################################################################################
Kazimierz O. Wrzeszczynski Ph.D.
# Cold Spring Harbor Laboratory, Cold Spring Harbor, NY 11724
# copyright 2011.
# Simple perl wrapper to run R wilcoxon analysis per gene.
# After this need to perform false discovery analysis
###################################################################################
#! /usr/bin/perl

$datafile = $ARGV[0]; #data input file.
#Datafile format.
#Chromosome    TSS.pos Sample  Gene    CNV     ExVal
#1     988913  1944.JB0795     ISG15   -0.0606 9.05
#1     988913  0688.JB0154     ISG15   -0.0265 9.76

open (FHFILE, $datafile) or die "could not open $normalfile\n";
while (<FHFILE>) {
   if ($_ =~ m/^\s+|^\#/){next;}
   $row = $_;
   chomp($row);
   @line = split(/\t/, $row);
   $gene=$line[3];
   $cnv=$line[4];
   $exval=$line[5];
   $sam=$line[2];
   $ampthresh=0.5; #sample/gene selection CNV threshold
   $delthresh= -0.25;
   if ($cnv < $delthresh){
         $normaldata{$gene}{$sam}=$exval; #normal is low CNV, tumor is high CNV;
   }
   if ($cnv >= $ampthresh){
         $tumordata{$gene}{$sam}=$exval; #tumor is high CNV;
   }

}
close FHFILE;

LINE: foreach $gid (keys %tumordata) {

   if ( (exists $tumordata{$gid}) && (exists $normaldata{$gid}) ){

   $datafile1=$gid."_tumor_data.txt";
   $datafile2=$gid."_normal_data.txt";

   open (FHOUT1, "> $datafile1");
   open (FHOUT2, "> $datafile2");

   print FHOUT1 "X\n";
   print FHOUT2 "X\n";

   foreach $point (keys %{$tumordata{$gid}}){
         printf FHOUT1 ("%3.6f\n", $tumordata{$gid}{$point}) if ($point =~ m/\d+/);
   }
   foreach $pointA (keys %{$normaldata{$gid}}){
```

```
            printf FHOUT2 ("%3.6f\n", $normaldata{$gid}{$pointA}) if ($pointA =~ m/\d+/);
    }

$fileoutR = $gid."_wilcox.r";

open (FHOUTB, ">$fileoutR") or die "could not open $fileoutR\n";

print FHOUTB "\#"."!/usr/bin/R\n\n";
print FHOUTB "tumor <- read.table(\"".$datafile1."\", header=TRUE)\n
normal <- read.table(\"".$datafile2."\", header=TRUE)\n
sink(\"".$gid."_wilcox_out.txt\", append=TRUE)\n
wilcox.test(normal\$X, tumor\$X)\$p.value\n
\n";
    close FHOUTB;


system ("Rscript ".$fileoutR);

} #ends if ( (exists $tumordata{$gid}) && (exists $normaldata{$gid}) ){

}#ends foreach $gid
#end of script
```
